# Routine machine quality assurance tests for a self‐shielded gyroscopic radiosurgery system

**DOI:** 10.1002/acm2.14589

**Published:** 2024-12-14

**Authors:** Yongsook C. Lee, Ranjini Tolakanahalli, D Jay Wieczorek, Minesh P. Mehta, Michael W. McDermott, Rupesh Kotecha, Alonso N. Gutierrez

**Affiliations:** ^1^ Department of Radiation Oncology Miami Cancer Institute Baptist Health South Florida Miami Florida USA; ^2^ Department of Radiation Oncology Herbert Wertheim College of Medicine Florida International University Miami Florida USA; ^3^ Department of Translational Medicine Herbert Wertheim College of Medicine Florida International University Miami Florida USA; ^4^ Department of Neurosurgery Miami Neuroscience Institute Baptist Health South Florida Miami Florida USA

**Keywords:** gyroscopic, machine quality assurance, radiosurgery, self‐shielded, Zap‐X

## Abstract

**Purpose:**

This report describes routine machine quality assurance (QA) (daily, monthly, and annual QA) tests for the Zap‐X^®^ Gyroscopic Radiosurgery^®^ platform.

**Methods:**

Following the recommendations of the American Association of Physicists in Medicine Task Group (AAPM TG)‐142 and Medical Physics Practice guideline (MPPG) 8.b, routine machine QA tests for the Zap‐X system were implemented. The implementation included (1) daily, monthly, and annual QA tests encompassing dosimetry, mechanical, safety and imaging tests, (2) QA methods of each test specific to the Zap‐X, (3) a tolerance value for each test, and (4) necessary QA equipment.

**Results:**

Baseline values and key results of daily, monthly, and annual QA tests are presented in this report. This report also discusses QA tests not adopted from TG 142 or MPPG 8.b (e.g., distance indicator) due to unique features of the Zap‐X system as well as additional QA tests added from the vendor's recommendations (e.g., self‐check) and from TG‐135 recommendations (e.g., monthly end‐to‐end testing) because of similarities between Zap‐X and CyberKnife systems.

**Conclusions:**

The comprehensive information on routine machine QA tests presented in this report will assist Zap‐X teams in other Neurosurgery centers or Radiation Oncology clinics in establishing and maintaining their QA programs until AAPM endorsed guidelines become available.

## 1 INTRODUCTION

The Zap‐X^®^ Gyroscopic Radiosurgery^®^ platform (Zap Surgical Systems, Inc., San Carlos, CA) is a novel radiation therapy device developed for frameless stereotactic radiosurgery (SRS) and stereotactic radiotherapy treatments of benign and malignant intracranial lesions as well as upper cervical spine lesions.^1^, ^2^, ^3^ There are several unique features to the Zap‐X system. First, the unit is self‐shielded.^4^ The design obviates the need for a costly and complex bunker.^2^ Second, the system houses an S‐band low energy (nominal energy of 3 megavoltage [MV]) linear accelerator (linac) with a source‐to‐axis distance (SAD) of 450 mm.^1^ The lower energy linac requires less shielding and produces beams with a smaller radiation penumbra compared to conventional linacs (6 MV or higher).^4^, ^5^, ^6^ The shorter SAD further helps to reduce the geometric penumbra. Third, radiation beam delivery follows a novel gyroscopic path.^7^ This unique geometry allows for more than 260 noncoplanar beams (slightly more than 2π steradian solid angle beam coverage)^7^ and consequently, equivalent, or better dosimetry compared to other dedicated SRS platforms.^8^, ^9^ To date, more than 20 units around the world are in clinical operation in Neurosurgery centers or Radiation Oncology clinics.

Early physics and dosimetry characterization work for the Zap‐X has been performed and published. These publications include commissioning of the system (treatment planning system [TPS] commissioning and beam data validation, output factor measurements, and absolute output calibration), shielding evaluation/analysis, and treatment planning studies.^2^, ^3^, ^4^, ^8^, ^9^, ^10^, ^11^ Excluding the vendor's brief description^1^ and a single clinic's experience in machine performance and stability,^12^ however, details on routine machine quality assurance (QA) tests have not yet been adequately addressed in the literature. While a fair number of the American Association of Physicists in Medicine Task Group (AAPM TG)‐142 QA tests can be directly adopted,^13^ several traditional QA tests and/or methodologies for conventional linacs are not applicable to the Zap‐X due to its unique design and aspects, and additional QA tests not listed in the TG‐142 report are required.^13^, ^14^, ^15^, ^16^ Therefore, the objective of this report is to describe the necessary routine machine QA (daily, monthly, and annual QA) tests for a Zap‐X gyroscopic radiosurgery system.

## 2 METHODS

### 2.1 Overview of the Zap‐X system

**FIGURE 1:**
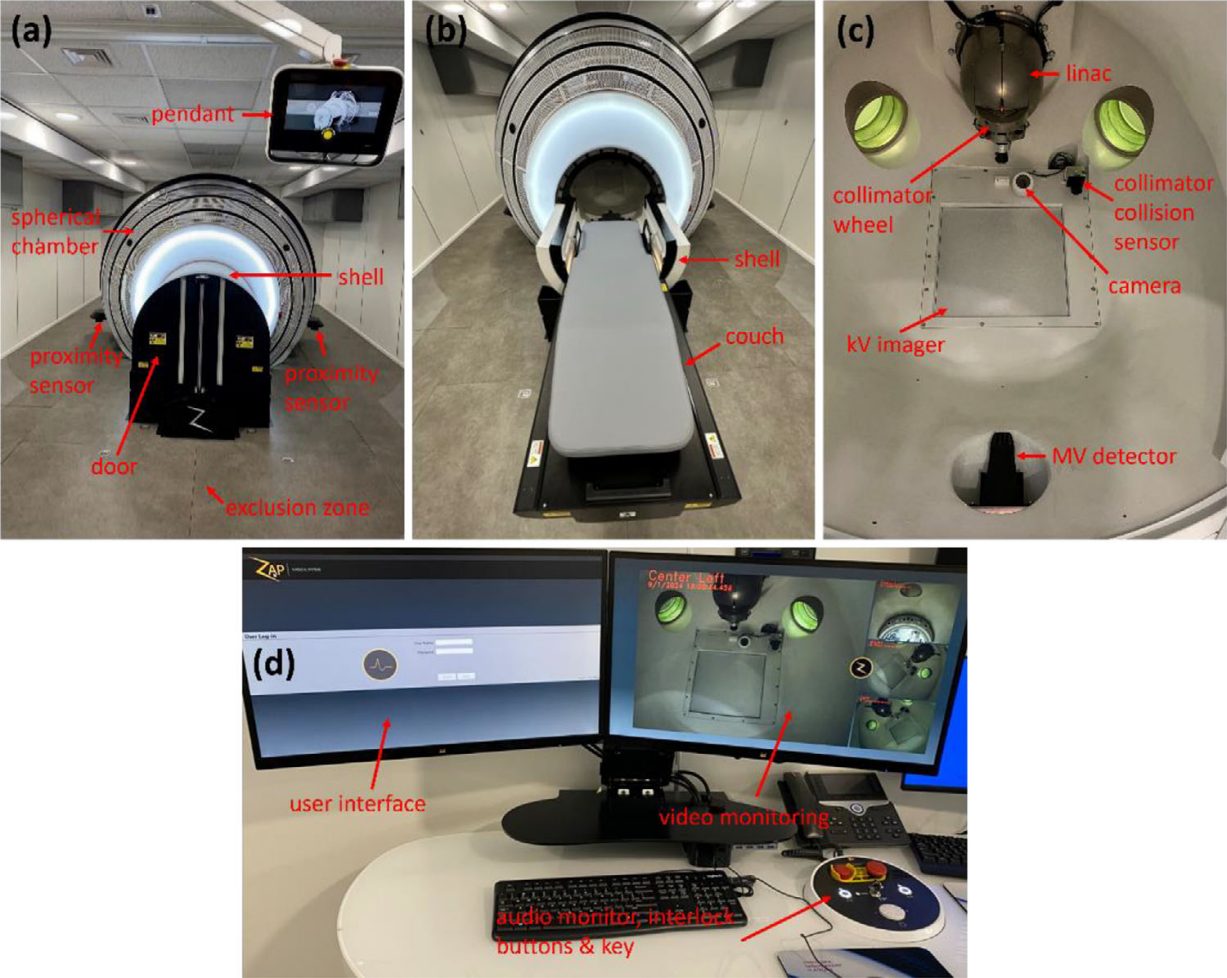
(a) Zap‐X system showcasing the spherical chamber, door, shell, pendant, and proximity sensors, (b) Zap‐X system shown in a ready to load patient state, (c) interior view of the spherical chamber with key components highlighted such as the collimating wheel, linac, MV detector, kV imager, collimator collision sensor, and patient view ports, and (d) treatment console, associated operator views and safety features. kV, kilovoltage; linac, linear accelerator; MV, megavoltage.

The Zap‐X is comprised of a shielded rotating spherical chamber, a pneumatic door with a rotary shell, a treatment couch, a linac, an internally mounted kilovoltage (kV) imaging system, an MV detector, safety systems and a pendant as shown in Figure 1.^10^, ^11^ The door and the shell move independently and create shielding for the couch. The couch has five degrees of freedom (yaw: left‐right; longitudinal: superior‐inferior; pitch: anterior‐posterior) and moves the planned isocenter of beams to the machine isocenter (isocentric treatment delivery).^3^, ^11^ The linac inside the spherical chamber moves in axial and oblique axes of independent rotations about the isocenter and produces 3 MV unflattened beams (d_max_ ∼ 7 mm) at a nominal dose rate of 1500 monitor unit (MU)/min.^11^ Circular collimation is achieved when the center of an opening (4, 5, 7.5, 10, 12.5, 15, 20, and 25 mm in diameter) in a tungsten‐shielded collimating wheel is aligned to the isocenter.^10^, ^11^ There is no optical distance indicator (ODI) nor light field in the linac with the exception of one laser in the linac head. The kV imaging system has a single kV x‐ray tube and a single kV imager for pretreatment patient alignment and intrafraction monitoring.^11^ The MV detector monitors exit dosimetry in real time.^11^ Multiple cameras inside the unit permit different views of the patient and the collimator collision sensor near the linac head/collimator prevents a collision. There is an exclusion zone around the unit which is detected by two external proximity sensors outside the spherical chamber. The console has other safety features. The pendant is used to move the couch to the isocenter as well as to close the door and the shell for treatment. The current software version for our system is DP‐1008.

### 2.2 Routine QA

Table 1 lists daily, monthly, and annual QA tests and their tolerances implemented and established at the time of commissioning in our clinic. Additionally, Table 1 includes QA equipment used for each test. The QA tests were based on the recommendations in AAPM TG‐142, TG‐198, TG‐135 and AAPM Medical Physics Practice Guideline (MPPG) 8.b reports as well as the description in Weidlich et al.^1^, ^13^, ^14^, ^15^, ^16^

**TABLE 1:** Daily, monthly, and annual QA tests, associated tolerances and necessary QA equipment for a Zap‐X gyroscopic radiosurgery system.

	Test		Tolerance	QA equipment
**Daily**	**Warm‐up**			
D1	Initialize		Successful completion	N/A
D2	Self‐check		Pass	N/A
D3	MU delivery: 6000 MU		Successful completion	N/A
	**Dosimetry tests**			
D4	Output constancy: 25 mm collimator		±3% from baseline	Zap‐X specific QA device, ionization chamber with a buildup cap, electrometer
D5	Dose rate		1395–1575 MU/min	In conjunction with output constancy check
	**Mechanical tests**			
D6	Collimator selection: all eight collimators		Functional	N/A
D7	Steel ball test: Winston‐Lutz test		±0.75 mm (all directions)	Head phantom with a steel ball cube
	**Safety tests**			
D8	CCTV cameras and monitors		Functional	N/A
	Audio monitor		Functional	N/A
	Radiation area monitor		Functional	N/A
	Interlocks: proximity sensors, HV off button, emergency stop buttons, key, and door		Functional	N/A
	**Imaging tests**			
D9	Positioning/repositioning		1 mm	In‐house device
D10	Imaging and treatment coordinate coincidence (single gantry position)		1 mm or center pixel ± 3.5 pixels	Zap‐X specific QA device, Zap‐X steel ball
	**Other test(s)**			
D11	Auxiliary parameters: water temperature, water flow, SF6 pressure, machine air pressure and compressor air pressure		±0.5°C, ±1.75 g/m, ±3 psi, ±10 psi and ±20 psi from baseline in order	N/A
**Monthly**	**Dosimetry tests**			
M1	Output constancy: 25 mm collimator		±2% from baseline	Zap‐X F‐bracket, Zap‐X specific QA device, ionization chamber, electrometer
M2	Beam energy constancy: 25 mm collimator		±1% from baseline	
M3	Backup monitor chamber constancy		±2% from nominal MU	In conjunction with output constancy check
M4	Beam profile constancy (symmetry and shape): 25 mm collimator		±2% from baseline	Zap‐X F‐bracket, film inserts, film
	**Mechanical tests**			
M5	Laser alignment		1.5 mm	Zap‐X F‐bracket, laser alignment tools
M6	Gantry angular accuracy		±1°	Zap‐X front pointer with the level bracket, digital level
M7	Treatment couch position indicators		±1 mm	Graph paper, solid water phantom
M8	E2E testing		0.95 mm	Head phantom with a film cube, films
	**Safety tests**			
M9	Collimator collision test		Functional	Large soft object
	**Imaging tests**			
M10	Imaging and treatment coordinate coincidence (four selected gantry positions)		1 mm or center pixel ± 3.5 pixels	Zap‐X specific QA device, Zap‐X steel ball
M11	Scaling		±1 mm	Zap‐X specific QA device, Zap‐X steel ball
M12	kV image quality	Spatial resolution	Baseline or better	kV imaging phantom, solid water phantom
		Contrast	Baseline or better	
		Uniformity	±5% from the average value for 5 ROIs	
		Noise	±5% from baseline	
**Annual**	**Dosimetry tests**			
A1	Beam profile constancy: all eight collimators		±2% from commissioning data	Water tank, microSilicon detector, reference detector, electrometer
A2	Beam quality (PDD10): 25 mm collimator		±1% from commissioning data	
A3	Output calibration: 25 mm collimator		±1% of nominal AAPM TG‐51 output	Water tank, ionization chamber, electrometer
A4	Output factors: all eight collimators		±2% from commissioning data	Water tank, microDiamond/microSilicon detectors, electrometer
A5	MU linearity: 25 mm collimator		±2% ≥5 MU	Water tank, ionization chamber, electrometer
A6	Output constancy versus gantry angle: 25 mm collimator		±1% of the value acquired at North Pole	Solid water phantom, ionization chamber, electrometer
	**Mechanical tests**			
A7	Positioning accuracy of the collimator wheel: all eight collimators		0.5 mm	Zap‐X F‐bracket, film inserts, films
A8	Coincidence of radiation and mechanical isocenter: star shots		±1 mm from baseline	Zap‐X star shot fixture, film
A9	Tabletop sag		2 mm from baseline	Head and body phantoms, solid water phantom, graph paper
	**Safety tests**			
A10	Radiation survey for leakage		2 mR/hr	Calibrated ionization chamber survey meter
A11	Emergency procedures/features		Successful completion/functional	N/A
	**Imaging tests**			
A12	Beam quality/energy		±5% from nominal kV_p_	Calibrated x‐ray detector
A13	Imaging dose		±3 mGy from baseline	Calibrated x‐ray detector
	**Other test(s)**			
A14	Complete E2E testing: from CT simulation to treatment delivery		1) 2D plane: ≥90% with gamma criteria of 2%/1 mm 2) Single point: ±5%	Head phantom with a film cube, film, detector array

Abbreviations: AAPM, American Association of Physicists in Medicine; CCTV, closed circuit television; CT, computed tomography; E2E, end to end; HV, high voltage; kV, kilovoltage; kV_p_, peak kilovoltage; MU, monitor unit; PDD, percent depth dose; QA, quality assurance; ROI, region of interest; SF6, sulfur hexafluoride; TG, task group.

#### 2.2.1 Daily QA

##### Warm‐up

**FIGURE 2:**
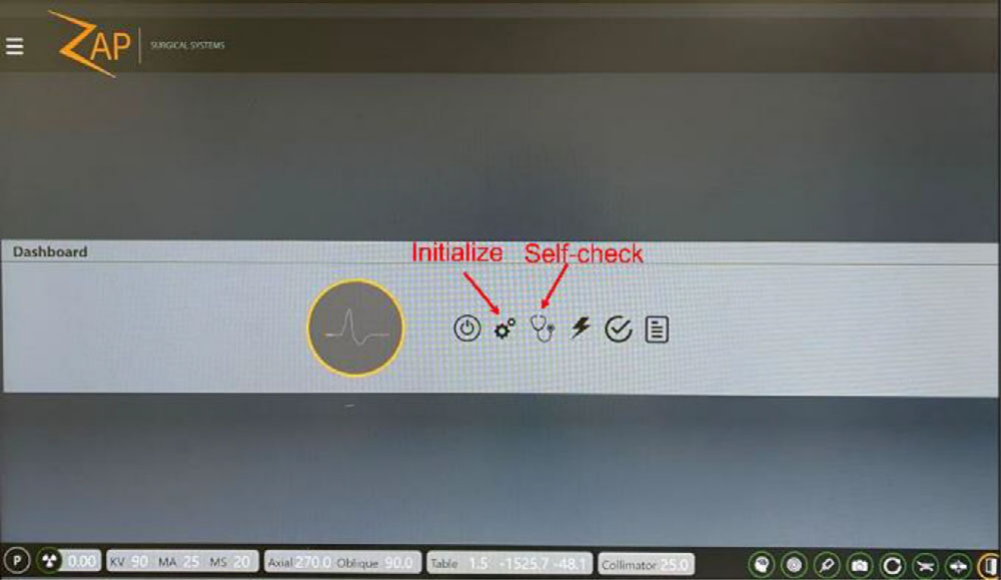
Treatment console operator view of the Zap‐X interface with the Initialize and Self‐check buttons highlighted.

*D1. Initialize*. Mandatory system initialization was performed in the user interface (UI) (Figure 2). The tolerance was set to successful completion.

*D2. Self‐check*. Upon initialization, self‐check was run in the UI (Figure 2). The self‐check is a pre‐programed test, checking full functionality of the system. The tolerance was set to pass.

*D3. MU delivery*. For linac warm‐up, 6000 MU were delivered. The Zap‐X has an open monitor chamber, and the recommendation from AAPM TG 135 was followed.^16^ The tolerance was set to successful completion.

##### Dosimetry tests

**FIGURE 3:**
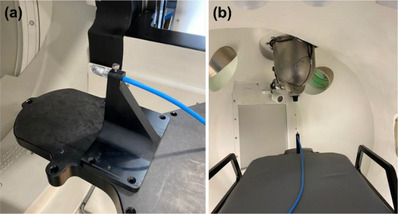
(a) Phantom setup on the table and (b) linac position for daily output constancy check. linac, linear accelerator.

*D4. Output constancy*. Daily output (cGy/MU) was measured using a Zap‐X specific QA device and an ionization chamber with a buildup cap. The device with an ionization chamber (PTW Semiflex 3D TN31021, PTW Freiburg, Freiburg, Germany) was mounted on the table (Figure 3a). The table was sent to the isocenter in the UI (Figure 3b). The linac was rotated to North Pole (linac facing straight down) (Figure 3b) and the 25 mm collimator was selected. The chamber position at the isocenter was verified using the laser through a camera monitor in the UI. With an electrometer bias set at –300 V (PTW UNIDOS), 500 MU were delivered 3–4 times and charges (nC) were collected. The temperature and pressure correction was applied to the average charge (nC) and a charge‐to‐dose conversion factor (cGy/[nC*MU]) was applied. The charge‐to‐dose conversion factor was obtained immediately following the absolute output calibration (A3 test in Section 2.2.3). The tolerance was set to ± 3% from the baseline.

*D5. Dose rate*. The nominal dose rate (1500 MU/min) was verified during the output constancy check. The tolerance range was set between 1395 and 1575 MU/min.

##### Mechanical tests

*D6. Collimator selection*. Collimator selection was verified by the correct operation of the collimator wheel rotation. Each collimator was selected through the UI and the selected collimator was cross‐checked against its appearance in the UI. The tolerance was set to functional.

**FIGURE 4:**
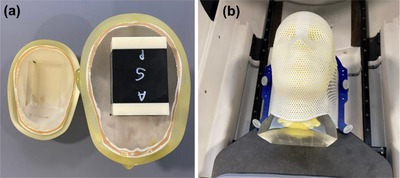
(a) Head phantom with a steel ball cube and (b) phantom setup on the table for the steel ball test.

*D7. Steel ball test*. The steel ball test is a pre‐programed test simulating the Winston‐Lutz test which verifies the coincidence between the radiation and mechanical isocenter for six beams at different gantry (axial/oblique) angles. For each beam, the phantom position is verified using kV imaging. The steel ball test was performed using an anthropomorphic head phantom with a steel ball cube (Figure 4a). The phantom was setup on the table (Figure 4b), the table was sent to the isocenter in the UI and the steel ball test was executed. Automated analysis was displayed in the UI after the test was completed. The tolerance was set to ± 0.75 mm for all directions. The action limit was set to ± 1 mm. The tolerance (± 0.75 mm) was established based on measurement data collected over the first 3 months.

##### Safety tests

*D8. Safety*. All safety features and interlock systems were verified. Safety checks encompass functionality checks of the cameras and monitors, audio monitor, and radiation area monitor as well as functionality checks of all interlock systems including proximity sensors, high voltage (HV) OFF button, emergency stop buttons (5 physical and 1 in the UI), key, and door. For interlock systems (Figure 1), it was verified that: (1) an emergency stop was activated when the exclusion zone was invaded during beam‐on, (2) beam turned off when the HV OFF button was pressed during beam‐on, (3) both the beam and motion stopped when one of the emergency stop buttons was pressed during beam‐on, and (4) the HV ON button was inactivated when the key was missing or turned to DISABLE on the console, or when the door was open. The tolerance was set to functional.

##### Imaging tests

*D9. Positioning/repositioning*. Detection and accurate corrections of positioning errors by the kV imaging system were verified using an in‐house device. A phantom (size: 5 cm × 6 cm × 10 cm) with three radiopaque markers (1 anterior and 2 lateral) and a flat mount were securely docked on the computed tomography (CT) overlay table. After the phantom position was marked on the mount, a CT simulation of the phantom was performed. A plan with a single isocenter intersecting the three markers was created in Zap‐X TPS solely for setup verification. The flat mount was docked on the treatment table. On the mount, the phantom was positioned 1 cm away from the initially marked position in left‐right and superior‐inferior directions, and a 1‐cm thick small piece was added underneath the phantom. The plan was loaded in Treatment mode and the table was sent to the isocenter using the pendant. Setup verification was performed using kV imaging, after which the plan was closed. The linac was rotated to North Pole in Daily QA mode, and with the laser on in the UI, the coincidence of the laser and the anterior marker on the phantom were verified through a camera monitor. Similarly, the linac was rotated to Patient Right and the coincidence of the laser and the lateral marker was checked. The tolerance was set to 1 mm (radius of the markers).

*D10. Imaging and treatment coordinate coincidence (single gantry position)*. Imaging and treatment coordinate coincidence was verified using a Zap‐X specific QA device and the Zap‐X steel ball. The device for the daily output check (Figure 3a) with the steel ball was mounted on the table. The table was sent to the isocenter in the UI and in this setup, the center of the steel ball should be at the isocenter. The linac was rotated to the North Pole. In service mode, a kV image of the steel ball was taken at the clinical setting (90 kVp, 25 mA and 20 ms). Electronic crosshairs were turned on and the coincidence between the center of the steel ball and electronic crosshairs was checked on the image. The distance (number of pixels) from the electronic crosshairs (kV imaging isocenter [*X*, *Y*]) to the center of the steel ball (treatment isocenter) was counted in ImageJ software (National Institutes of Health and Laboratory for Optical and Computational Instrumentation, version 1.54j). The kV imaging isocenter coordinate (*X*, *Y*) was found in the configuration file. The tolerance was set to 1 mm or center pixel ±3.5 pixels.

##### Other test(s)

*D11. Auxiliary parameters*. After the system was up, water temperature, water flow, sulfur hexafluoride (SF6) pressure, and air pressure for the machine and the compressor were checked. The tolerances for water temperature, water flow, SF6 pressure, machine air pressure, and compressor air pressure were set to ± 0.5°C, ± 1.75 g/m, ± 3 psi, ± 10 psi and ± 20 psi from the baseline, respectively.

#### 2.2.2 Monthly QA

##### Dosimetry tests

**FIGURE 5:**
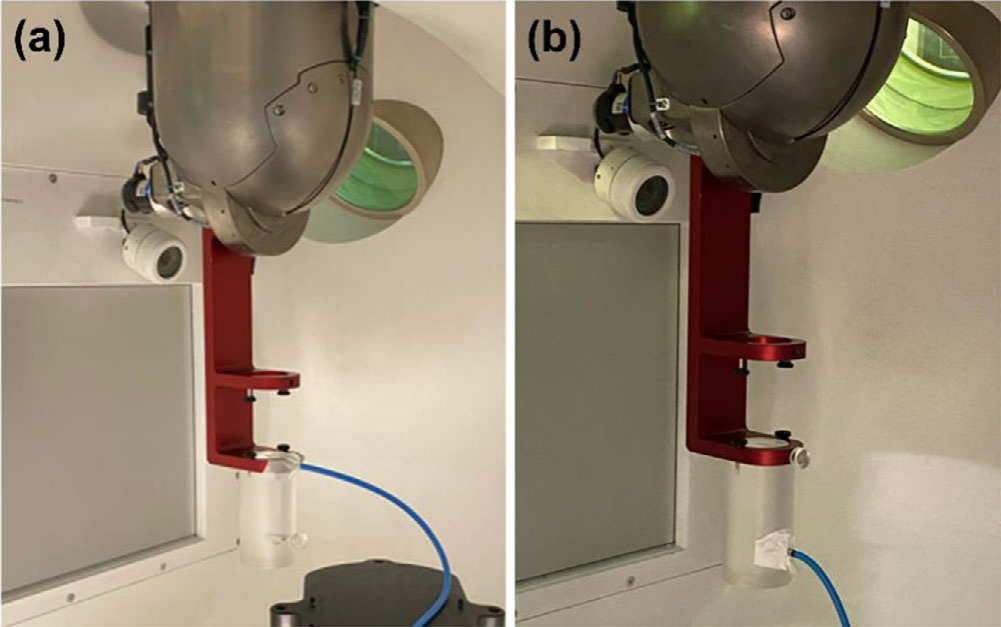
Phantom setup for (a) monthly output constancy check and (b) energy constancy check.

*M1. Output constancy*. Monthly output (cGy/MU) was measured using the Zap‐X F‐bracket, a Zap‐X specific QA device and an ionization chamber. The acrylic cylindrical phantom provided by the vendor was attached to the F‐bracket which was then mounted under the linac. The linac was rotated to North Pole (Figure 5). An ionization chamber (PTW Semiflex 3D TN31021) was inserted into the top hole of the phantom (Figure 5a) such that the center of the chamber was positioned at d_max_ (water equivalent) in SAD setup (i.e., source to the chamber distance of 450 mm). An acrylic plug was inserted into the bottom hole (Figure 5a). In the UI, the 25 mm collimator was selected. With an electrometer bias set at –300 V (PTW UNIDOS), 500 MU were delivered 3–4 times and charges (nC) were collected. The temperature and pressure correction were applied to the average charge (nC) and a charge‐to‐dose conversion factor (cGy/[nC*MU]) was also applied. The charge‐to‐dose conversion factor was obtained immediately after the absolute output calibration (A3 test in Section 2.2.3). The tolerance was set to ± 2% from the baseline.

*M2. Beam energy constancy*. In the same setup used for the output constancy check, the chamber and the acrylic plug were repositioned to the bottom hole of the phantom at a depth of 10 cm (water equivalent) and to the top hole, respectively (Figure 5b). The 25 mm collimator was confirmed in the UI, and 500 MU were delivered 3–4 times and charges (nC) were collected. The ratio of [average charge]_10cm_ to [average charge]_dmax_ was calculated for beam energy check. The ratio was established immediately after the absolute output calibration. The tolerance was set to ± 1% from the baseline.

*M3. Backup monitor chamber constancy*. During the output constancy check, secondary MU was monitored. The tolerance was set to ± 2% from MU set on the console (nominal MU).

**FIGURE 6:**
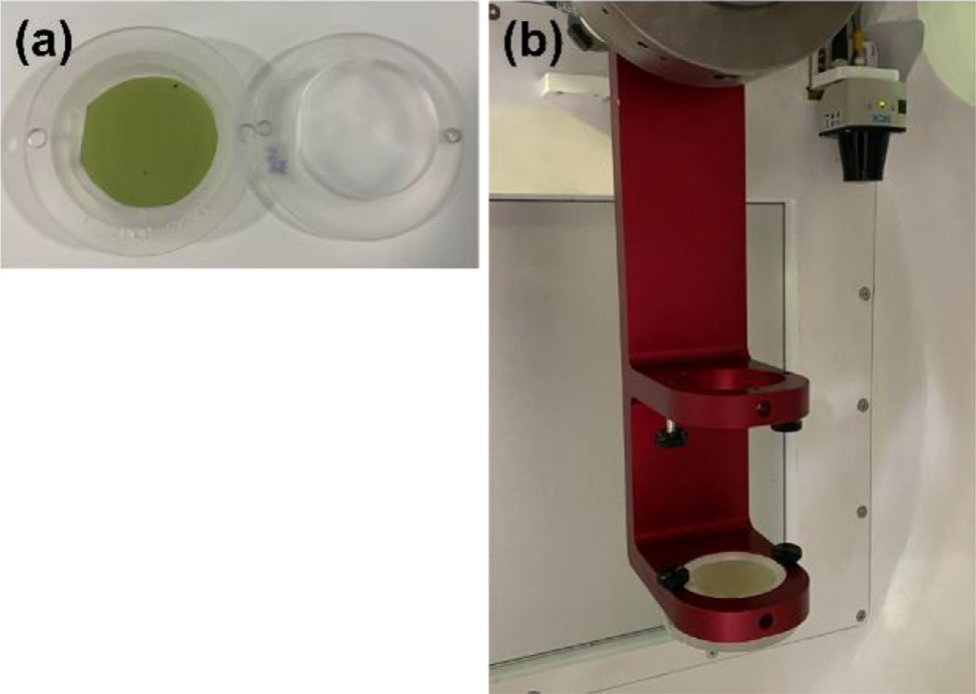
(a) Film and film inserts and (b) F‐bracket with the film inserts for beam profile constancy check.

*M4. Beam profile constancy (symmetry and shape)*. Beam profiles for the 25 mm collimator was measured using the F‐bracket, film inserts and a film. A radiochromic film (EBT3, Ashland LLC, Bridgewater, NJ, USA) was placed in acrylic inserts (Figure 6a) and the inserts were attached in the F‐bracket which was then mounted under the linac (Figure 6b). The top insert has a 7‐mm water equivalent buildup, and therefore, the film was placed at d_max_ in SAD setup. In the North Pole position, the 25 mm collimator was selected, and 500 MU were delivered. After 24 h, the film was scanned with 300 dots per inch (dpi) using a flatbed reflective scanner (EPSON 10000XL, Seiko Epson Corp., Nagano, Japan). Film analysis was performed in FilmQA Pro™ software (Ashland LLC) to calculate symmetry (%) for wheel (parallel to the collimator wheel) and ortho (perpendicular to the wheel) planes using the embedded AAPM TG‐45 protocol: a maximum permissible percentage deviation of the “left‐side” dose from the “right‐side” dose of a beam profile at 80% of the full width at half maximum (FWHM) points.^17^ The tolerance was set to ± 2% from the baseline.

##### Mechanical tests

**FIGURE 7:**
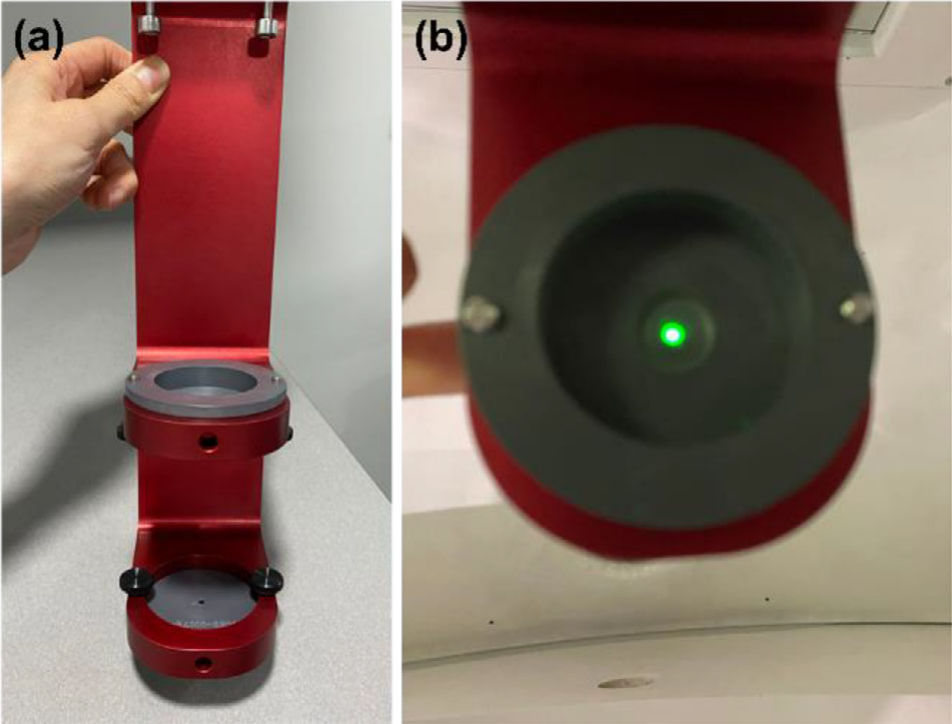
(a) Laser alignment tools attached to the F‐bracket and (b) laser alignment check.

*M5. Laser alignment*. The laser in the linac head is aligned to the isocenter. The laser is not used for patient setup but can be used for QA purposes. A laser alignment check was performed using the F‐bracket and laser alignment tools. Two laser alignment tools with a 3‐mm diameter hole were attached to the F‐bracket (upper and lower) (Figure 7a) and the F‐bracket was mounted under the linac. In the North Pole position, the 4 mm collimator was selected, and the laser was turned on in the UI. Laser alignment through upper and lower holes of the alignment tools was verified (Figure 7b). The tolerance was set to 1.5 mm (radius of the hole in the laser alignment tools).

**FIGURE 8:**
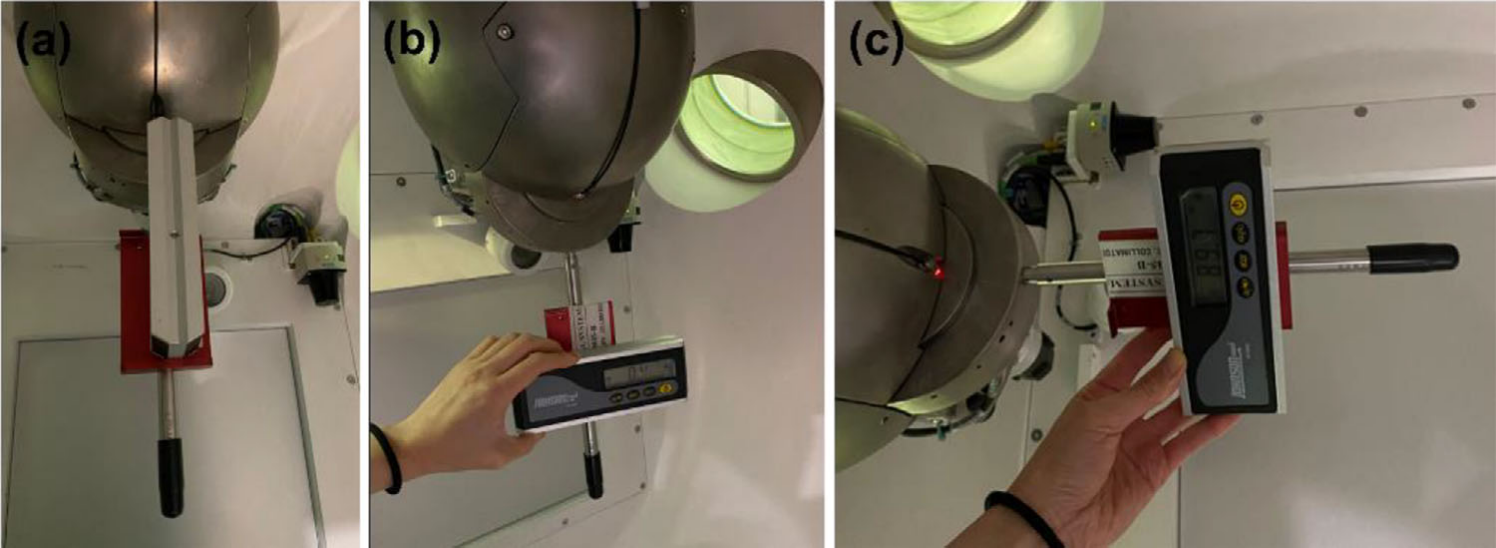
(a) Home, (b) North Pole, and (c) Patient Right positions of the linac for gantry angular accuracy check. linac, linear accelerator.

*M6. Gantry angular accuracy*. Gantry angular accuracy was checked using the Zap‐X front pointer with the level bracket and a digital level. This test was done for five selected linac positions: Home, North Pole, Patient Right, South Pole, and Patient Left. In the Home position, the linac is tilted posteriorly by 45 degrees from the North Pole position (Figure 8a). In North Pole and South Pole positions, the linac faces straight down (0 degree) and straight up (0 degree), respectively (Figure 8b). In Patient Right and Patient Left positions, the linac is angled by 90 degrees from the North Pole position to patient's right and left in a supine position, respectively (Figure 8c). These angles (45°, 0°, and 90°) are inclinations (physical tilts) of the linac and do not represent axial or oblique angles for each linac position. In the UI, the 25 mm collimator was selected. The front pointer with the level bracket was inserted into the collimator (Figure 8). In each linac position, a digital level was placed on the bracket and the angle was measured (Figure 8). In all positions except Home, the front pointer was rotated all around by 360 degrees to find the largest angle. Expected angles for Home, North Pole, Patient Right, South Pole, and Patient Left positions were 45°, 0°, 90°, 0°, and 90°, respectively. The tolerance was set to ±1°.

*M7. Treatment couch position indicators*. Treatment couch position indicators were verified using a graph paper and a solid water phantom. A graph paper was taped on the couch head and the couch was sent to the isocenter in the UI. In the North Pole position of the linac, using the laser, the initial couch position was marked on the graph paper. The couch was shifted by 50 mm laterally (patient left and right, each) in service mode. The new couch position was marked using the laser and the difference between the two marks was measured on the graph paper. Similarly, the couch was moved by 50 mm longitudinally (patient superior and patient inferior, each), and the difference was measured on the graph paper. Next, a phantom (size: 30 cm × 30 cm × 3 cm, Virtual Water^®^, CNMC, Nashville, TN, USA) with a graph paper taped was vertically placed on the couch around the isocenter and the couch was sent to the isocenter in the UI. In the Patient Right position of the linac, using the laser, the initial couch height was marked on the graph paper. The couch was dropped and raised by 50 mm (patient anterior and patient posterior, each). The new couch height was marked using the laser and the difference between the two marks was measured on the graph paper. After the 50‐mm shift in each direction from the isocenter (0, 0, 0), couch coordinates (*X*, *Y*, *Z*) for table left (patient right), table right (patient left), table out (patient superior), table in (patient inferior), table down (patient anterior), and table up (patient posterior) were (–50, 0, 0), (50, 0, 0), (0, –50, 0), (0, 50, 0), (0, 0, –50), and (0, 0, 50), respectively. The tolerance was set to ± 1 mm.

**FIGURE 9:**
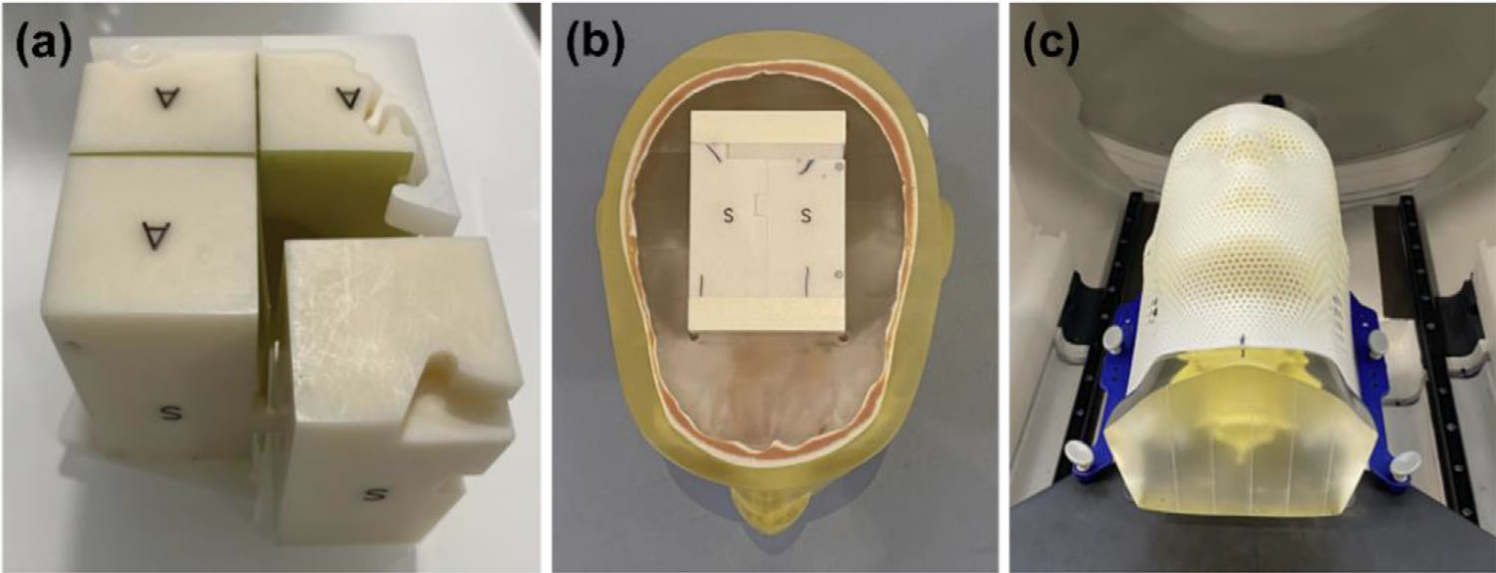
(a) Film cube with two orthogonal films, (b) head phantom with the film cube, and (c) phantom setup on the table for end‐to‐end testing.

*M8. End‐to‐end (E2E) testing*. Like the CyberKnife (CK) system (Accuracy, Sunnyvale, CA, USA), E2E testing was incorporated in the Zap‐X system to verify overall radiation delivery accuracy. Using E2E testing, differences among the radiation center (center of the beams), imaging isocenter and mechanical isocenter (center of the target) in anterior‐posterior, left‐right, and superior‐inferior directions are evaluated. E2E testing was performed using an anthropomorphic head phantom with a film cube and films. Two orthogonal EBT3 films were inserted in a film cube (Figure 9a), the film cube was inserted in the phantom (Figure 9b) and the phantom was setup on the table (Figure 9c). A treatment plan created in Zap‐X TPS using the 25 mm collimator (single isocenter, 30 beams) was loaded in Treatment mode, the table was sent to the isocenter using the pendant and the plan was delivered. After 24 h, the films were scanned with 300 dpi on a scanner (EPSON 10000XL). Film analysis was performed using the Accuracy End‐to‐End Film Analysis^®^ software. Film analysis can be done using the FilmAnalysisApp.exe (Zap Surgical Systems, Inc.). The tolerance was set to 0.95 mm.^16^

##### Safety tests

**FIGURE 10:**
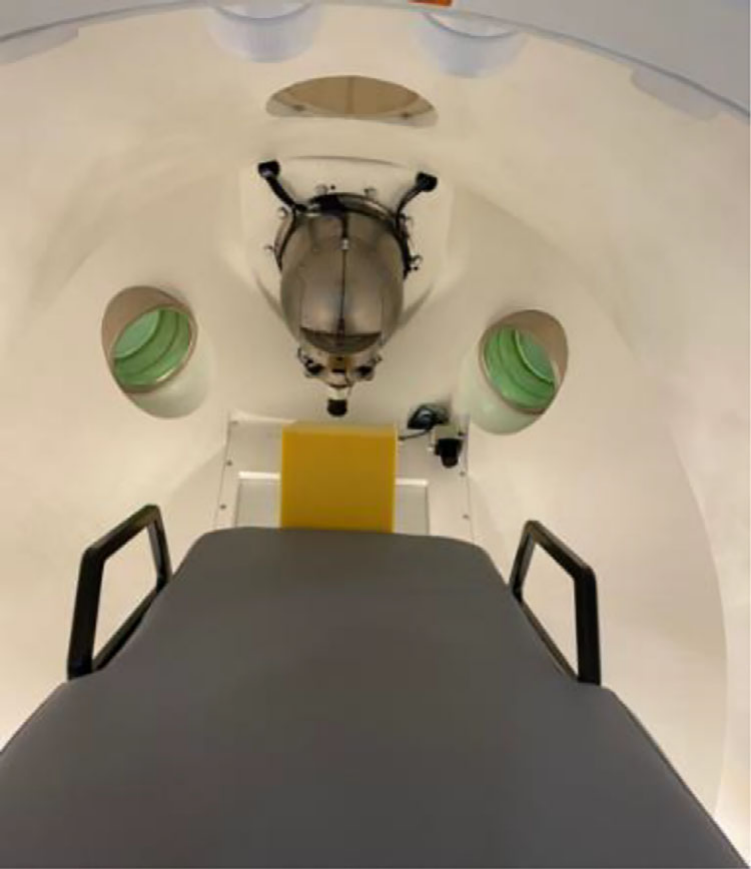
Collimator collision test using a soft object on the table.

*M9. Collimator collision test*. A collimator collision test was performed using a large and soft object. The linac was rotated to the Home position. The object was placed at the end of the table, and the table was manually pushed in until the object was very close to the collimator (Figure 10). It was checked if the collision indicator was turned on (green to orange) in the UI and table motion was inactivated. This test is similar to the laser guard interlock test recommended in TG 142. The tolerance was set to functional.

##### Imaging tests

*M10. Imaging and treatment coordinate coincidence (four selected gantry positions)*. The D10 test was performed for Home, Patient Right, South Pole, and Patient Left positions of the linac. The tolerance was set to 1 mm or center pixel ± 3.5 pixels.

*M11. Scaling*. The dimensions of the images scaled to isocenter were verified using a Zap‐X specific device and the Zap‐X steel ball. The device for the daily output check with the steel ball (diameter: 3/4″ [19.05 mm]) was mounted on the table (same setup as the D10 test). The table was sent to the isocenter and the linac was rotated to North Pole in the UI. In service mode, a kV image of the steel ball was taken at the clinical setting (90 kVp, 25 mA and 20 ms). Using ImageJ software, the number of pixels for the diameter of the steel ball was counted. The diameter of the steel ball was determined from the pixel size × the number of pixels. The pixel size at isocenter was 0.2885 mm/pixel (= 0.4 mm/pixel [pixel size at kV imager] × 927.15 mm [distance from kV source to isocenter]/1285.29 mm [distance from kV source to kV imager]). Measurements were repeated for Home, Patient Right, South Pole and Patient Left positions of the linac. The tolerance was set to ±1 mm.

**FIGURE 11:**
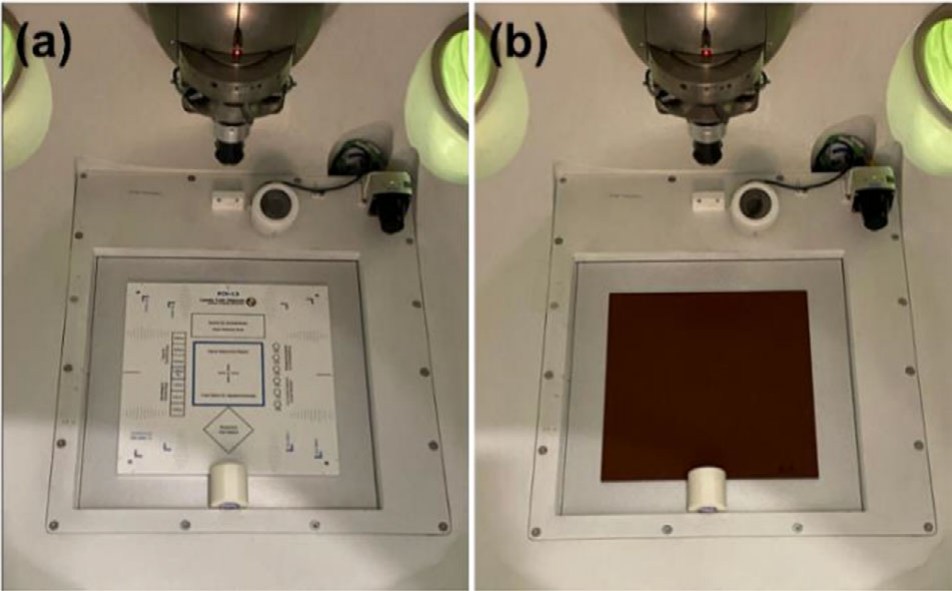
Setup with (a) kV imaging phantom and (b) slab phantom for kV image quality check. kV, kilovoltage.

*M12*. kV *image quality*. kV image quality (spatial resolution, contrast, uniformity, and noise) was verified using a kV imaging phantom and a solid water phantom. The linac was rotated to Home. The PIX‐13 phantom (Leeds Test Objects Ltd., Boroughbridge, North Yorkshire, UK) was placed on the kV imager (Figure 11a). In service mode, a kV image of the PIX‐13 phantom (Image 1) was taken at the clinical setting (90 kVp, 25 mA and 20 ms). The phantom was removed, a uniform slab phantom (size: 30 cm × 30 cm × 0.5 cm, Virtual Water, CNMC) was placed on the imager (Figure 11b) and a kV image of the slab phantom (Image 2) was taken. Image analysis was performed in ImageJ software. On Image 1, spatial resolution (line pairs/mm) and contrast (number of visibly resolved circles or dynamic wedges) were determined, and the tolerances for spatial resolution and contrast were set to the baseline or better (i.e., at least the baseline). To determine uniformity and noise, the method described in Chang et al was followed.^18^ On Image 2, a region of interest (ROI) of a 1 × 1 cm^2^ square was placed at the center as well as on the left, right, top and bottom (7.5 cm off‐center), and the value (Mean) for each of the five ROIs was taken. The tolerance for uniformity was set to ± 5% from the average values for five ROIs. On Image 2, an ROI of a 5 × 5 cm^2^ square was placed at the center and Mean and standard deviation (SD) for the ROI were taken. Noise was determined as SD/Mean. The tolerance for noise was set to ± 5% from the baseline.

#### 2.2.3 Annual QA

##### Dosimetry tests

**FIGURE 12:**
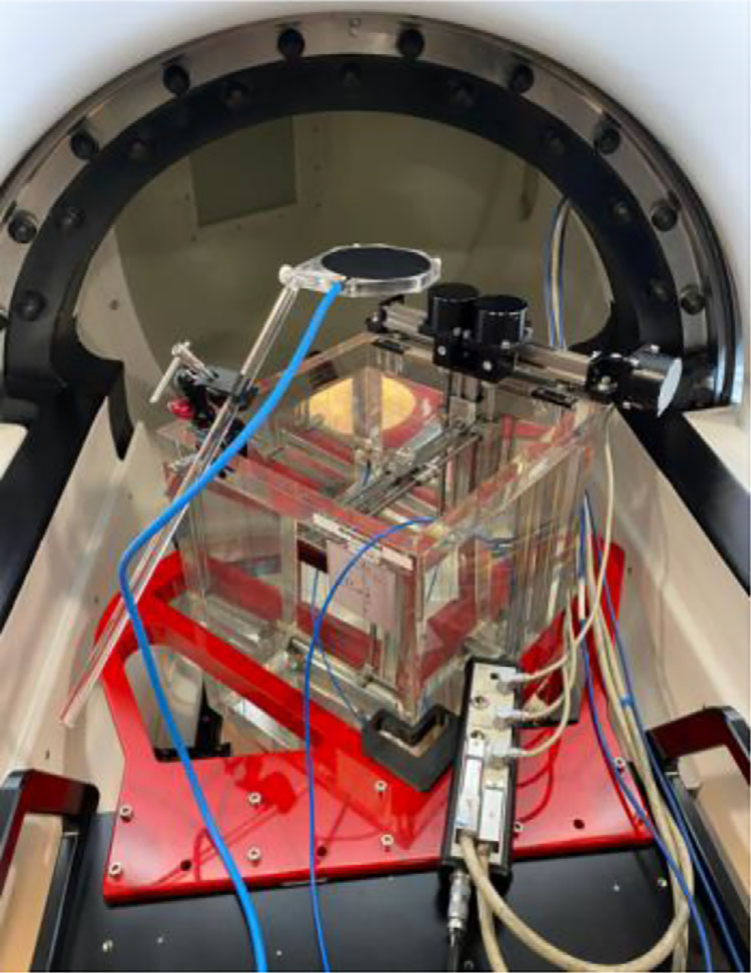
The PTW water tank set up for beam profile constancy check. The red bracket is attached to the treatment table and supports the water tank during beam scanning.

*A1. Beam profile constancy*. Beam profiles for all eight collimators were measured using a water tank, a microSilicon detector and a reference detector. The linac was rotated to North Pole, and the 25 mm collimator was selected in the UI. The end of the table was removed, the PTW bracket was attached to the table, and a water tank (PTW MP3‐MS water phantom T41058) was setup in the bracket (Figure 12). After the tank was leveled, the tank was filled with water, the table was manually pushed in, and a source to surface distance (SSD) of 450 mm was set using the front pointer. A microSilicon detector (PTW TN60023) and a reference ionization chamber (PTW T‐REF TN34091) were mounted in the water tank (Figure 12). With an electrometer bias set at –400 V for the reference detector, profiles for wheel and ortho planes were scanned at depths of 0.7  (d_max_), 5, 10, 20, and 25 cm using PTW MEPHYSTO software (version 4.6.5). After post‐processing (smoothing), symmetry and flatness were measured from the profiles at d_max_ for the 25 mm collimator. Symmetry was defined as (Dose_right_ [%]–Dose_left_ [%])_max_, and flatness as (Dose_max—_Dose_min_)/2 within 80% of FWHM points. Measurements were repeated for the rest of the collimators. From profiles, field size (at d_max_) and off‐axis factors are annually checked. The tolerance for beam profiles was set to ± 2% from the commissioning data.

*A2. Beam quality*. In the same SSD setup, using the same QA equipment, the percentage depth dose (PDD) was measured. PDD at a depth of 10 cm for the 25 mm collimator was determined as beam quality. The tolerance was set to ± 1% from the commissioning data.

*A3. Output calibration*. In the same SSD setup, output calibration was performed following the AAPM TG‐51 protocol. An ionization chamber (PTW Semiflex 3D TN31021) was mounted in the PTW water tank and was positioned at a depth of 5 cm. In the UI, the 25 mm collimator was selected. With electrometer bias set at –300, –150, and 300 V each (PTW UNIDOS), 500 MU were delivered 3–4 times and charges (nC) were collected at each bias. The temperature and pressure correction ($P_{TP}$,), ion recombination ($P_{ion})$, polarity effects ($P_{Pol}$), electrometer correction factor ($P_{ele}$), quality conversion factor ($k_{Q}=1$) and absorbed dose to water calibration factor ($N_{D,w}^{Q}$) were multiplied by the average charge ($M_{raw}\;\left[-300V\right]$). PDD at a depth of 5 cm and an inverse square correction ([450+7 mm]^2^/[450 mm]^2^) were then applied to calibrate the machine output to 1 cGy/MU at d_max_ (7 mm) in SAD setup. Details on absolute dose measurements are found in Sorensen et al.^10^ The tolerance was set to ± 1% of the nominal output (1 cGy/MU).

*A4. Output factors*. In SAD setup, output factors were measured using the PTW water tank and microSilicon and microDiamond detectors. Using the front pointer, SAD of 450 mm was set in the water tank. A microSilicon detector (PTW TN60023) was mounted in the water tank and was positioned at d_max_. For each collimator, 500 MU were delivered 3–4 times and charges (nC) were collected using an electrometer (PTW UNIDOS). Output factors were determined as a ratio of [average charge]_4‐20 mm_ to [average charge]_25 mm_. Measurements were repeated using a microDiamond detector (PTW TN60019). Detector specific field output correction factors for Zap‐X are not available in the literature yet and therefore, were not applied to the measured output factors. The tolerance was set to ± 2% from the commissioning data.

*A5. MU linearity*. In SAD setup, MU linearity was measured using the PTW water tank and an ionization chamber. An ionization chamber (PTW Semiflex 3D TN31021) was mounted in the water tank and was positioned at d_max_. In the UI, the 25 mm collimator was selected. With an electrometer bias set at –300 V, 10, 50, 100, 500, and 1000 MU were delivered in order and charges (nC) were collected. A linear regression line was plotted and a deviation from the linear regression line for each delivered MU was calculated. The tolerance was set to ± 2%.

**FIGURE 13:**
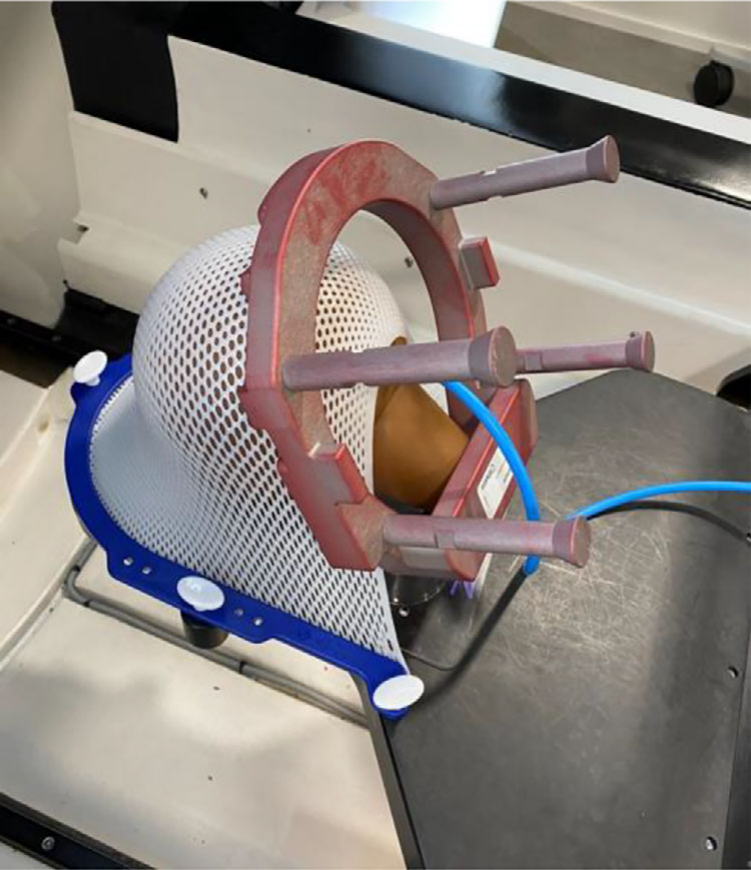
Phantom setup for output constancy check versus gantry angle.

*A6. Output constancy versus gantry angle*. Output as a function of gantry angle was checked using a solid water spherical phantom and an ionization chamber. Due to the difficulty of accurate phantom setup at isocenter, a treatment plan was used for setup verification. A CT simulation of the Leksell Gamma Knife dosimetry phantom (Elekta AB, Stockholm, Sweden) with an ionization chamber (PTW TN31010) was performed (Figure 13). In this setup, the ionization chamber was positioned at the center of the spherical phantom. A plan with a single isocenter located at the center of the ionization chamber was created in Zap‐X TPS. The phantom with the chamber was setup on the table (Figure 13). The plan was loaded in Treatment mode and the table was sent to the isocenter using the pendant. Setup verification was performed using kV imaging, after which the plan was closed. The linac was rotated to North Pole in Daily QA mode and with an electrometer bias set at –300 V (PTW UNIDOS), 500 MU were delivered 3–4 times and charges (nC) were collected. Measurements were repeated for Patient Right, South Pole, and Patient Left positions of the linac. A variation (%) from the average charge for North Pole was calculated for each linac position. The tolerance was set to ± 1% of the value acquired at North Pole.

##### Mechanical tests

**FIGURE 14:**
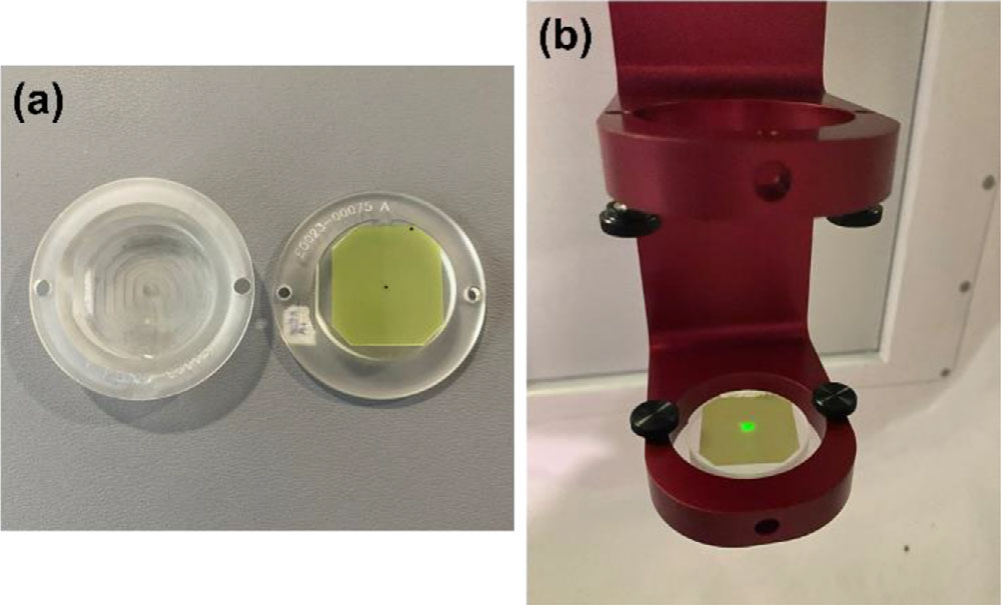
(a) Film taped on the film insert and (b) marking on the film using the laser for collimator wheel positioning accuracy check.

*A7. Positioning accuracy of the collimator wheel*. Positioning accuracy of the collimator wheel was verified for all eight collimators using the F‐bracket, film inserts, and films. The F‐bracket was mounted under the linac, the linac was rotated to North Pole, and laser alignment was checked using laser alignment tools following the M5 test (Section 2.2.2). An EBT3 film was taped to the bottom film insert (Figure 14a), and the insert was attached to the F‐bracket. Using the laser in the linac, a small dot (isocenter) was marked on the film (Figure 14b). The top insert with a 7‐mm water equivalent buildup was attached to the F‐bracket such that the film was placed at d_max_ in SAD setup. In the UI, the 25 mm collimator was selected, and 500 MU were delivered. Measurements were repeated for the rest of the collimators. After 24 h, the films were scanned with 300 dpi on a scanner (EPSON 10000XL). Film analysis was performed using RIT software (Radiological Imaging Technology, Inc., Colorado Springs, CO, USA) to evaluate the coincidence between the center of the dot (machine isocenter) and the radiation center (center of the collimator) for each collimator. The tolerance was set to 0.5 mm.

**FIGURE 15:**
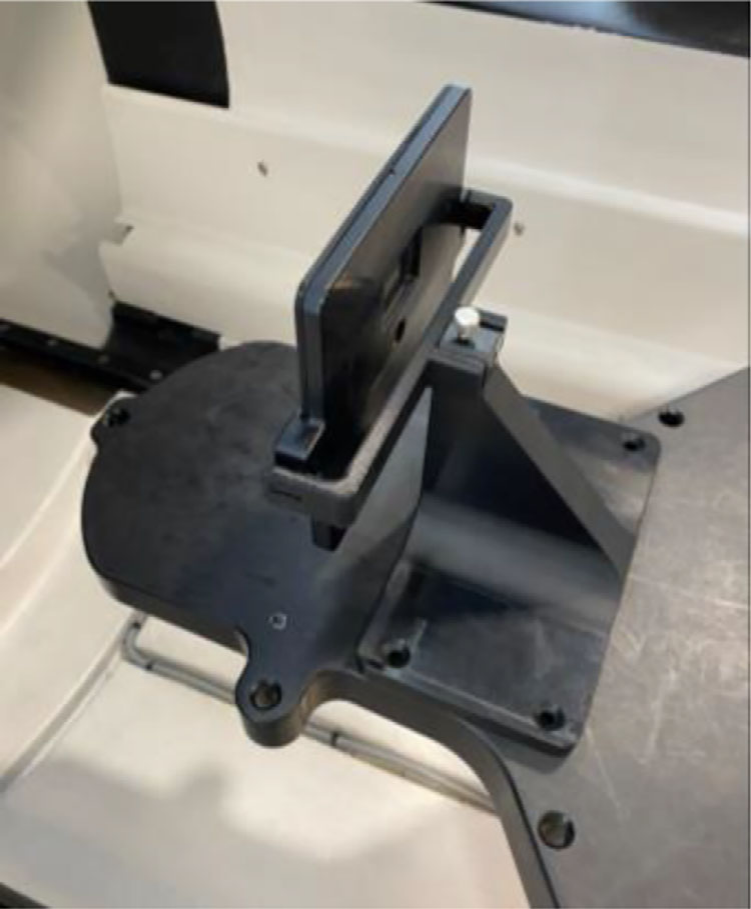
Setup for start shot measurement.

*A8. Coincidence of radiation and mechanical isocenter*. The coincidence of the radiation and mechanical isocenter was checked using the star shot technique. An EBT3 film was inserted in the Zap‐X star shot fixture and the fixture was mounted on the table (Figure 15). The table was sent to the isocenter in the UI and the film location was confirmed using the laser through a camera monitor in the UI. Five beam angles with axial and oblique angle combinations (axial angles: 270°, 342°, 54°, 126°, 198°; oblique angle: 90°) were selected such that the linac rotated around the fixture every 72°. The 5 mm collimator was selected, and 500 MU were delivered for each beam. After 24 h, the film was scanned with 300 dpi on a scanner (EPSON 10000XL). Film analysis was performed in RIT software to find a minimum tangent circle radius. Due to the risk of collision, star shots at different oblique angles were not performed. The tolerance was set to ± 1 mm from the baseline.

**FIGURE 16:**
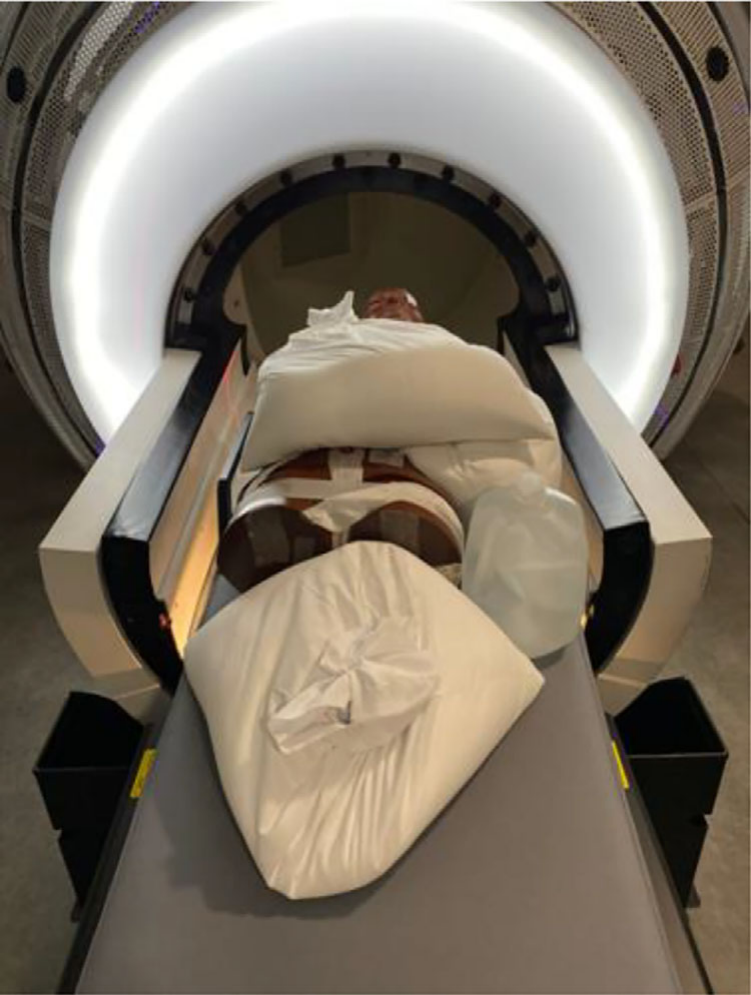
Setup for tabletop sag measurement.

*A9. Tabletop sag*. Tabletop sag was measured using anthropomorphic head and body phantoms, a solid water phantom and a graph paper. The head and body phantoms were setup on the table simulating patient setup. Three sandbags and a 1‐gallon water bottle were added onto the phantom's abdomen and legs with a total combined weight of 202.5 lbs (Figure 16). The table was manually pushed to the isocenter. The linac was rotated to Patient Right. A phantom (size: 30 cm × 30 cm × 3 cm, Virtual Water, CNMC) with a graph paper taped was vertically placed around the isocenter next to the head phantom. Using the laser in the linac, the table height was marked on the graph paper (same method as for the M7 test in Section 2.2.2). Without the head and body phantoms, sandbags and water bottle, the table height was marked on the graph paper. The difference between the two marks was measured on the graph paper. The tolerance was set to 2 mm from the baseline.

##### Safety tests

**FIGURE 17:**
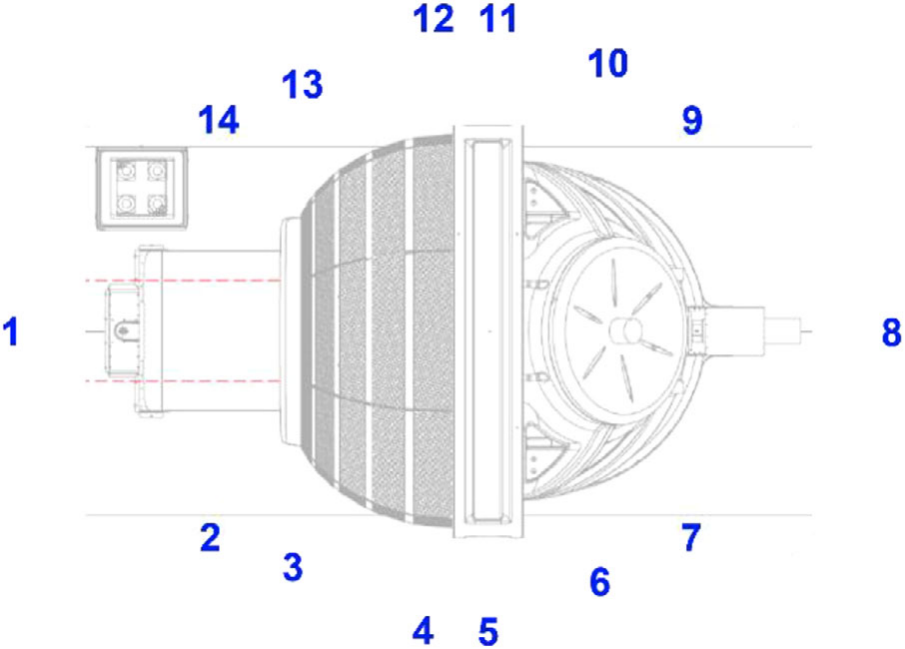
Locations around the Zap‐X unit for leakage radiation measurements.

**FIGURE 18:**
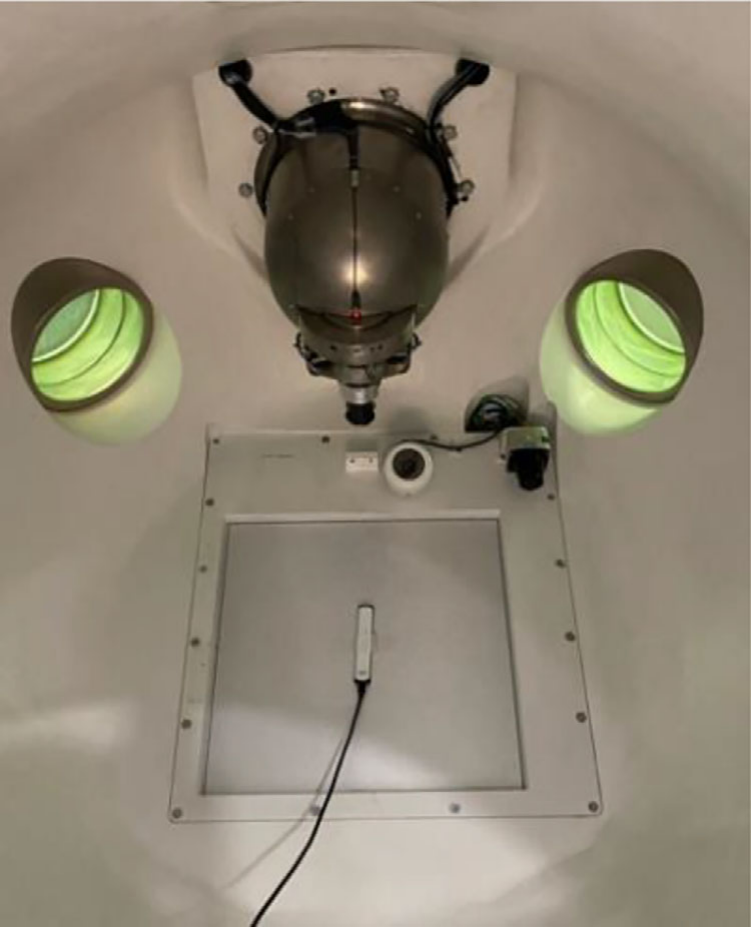
X‐ray detector setup for kV beam quality/energy measurement. kV, kilovoltage.

*A10. Radiation survey for leakage*. A radiation survey was conducted in a full scatter condition around the unit using a calibrated ionization chamber survey meter. Anthropomorphic head and body phantoms were setup on the table (same setup as for the A9 test) and the table was sent to the isocenter. The linac was rotated to Home and the 25 mm collimator was selected. While the beam was on, the Fluke 451 survey meter (Fluke Biomedical LLC, Everett, WA, USA) was placed at the edge of the exclusion zone in 14 different locations designated in Figure 17, 18 and leakage radiation (mR/hr) was measured. Measurements were repeated for the North Pole, Patient Right, South Pole, and Patient Left positions of the linac. The tolerance was set to 2 mR/hr.

*A11. Emergency procedures/features*. In‐house emergency procedures were reviewed and performed with all clinical personnel involved. Emergency features such as the beam off, emergency stops, emergency power off, manual door release (and manual shell release in the version of DP‐1010) were tested. Successful completion of the procedures was required for all involved and the tolerance for emergency features was set to functional.

##### Imaging tests

*A12. Beam quality/energy. Beam quality/energy was measured using a calibrated x‐ray detector*. The linac was rotated to Home. The x‐ray detector (RaySafe Xi, Unifors RaySafe AB, Hovas, Sweden) was taped in the center of the kV imager (Figure 18) and a kV image was acquired 3–4 times in service mode at the clinical setting (90 kVp, 25 mA and 20 ms) with energy (kV) measured in each acquisition. The tolerance was set to ± 5% from the nominal kV_p_.

*A13. Imaging dose*. Using the same setup as for beam quality/energy measurements, imaging dose (µGy) was measured while a kV image was taken. The tolerance was set to ± 3 mGy from the baseline.^19^

##### Other test(s)

**FIGURE 19:**
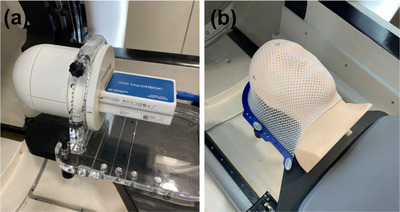
(a) Detector array setup for 2D plane measurement and (b) head phantom setup for point dose measurement (complete end‐to‐end testing).

*A14. Complete E2E testing*. Following the MPPG 8.b recommendation,^15^ complete E2E testing was performed using an anthropomorphic head phantom with a film cube, a film, and a detector array. Following our clinical workflow, a CT simulation of the MAX HD 2.0 SRS phantom (Standard Imaging Inc, Middleton, WI, USA), magnetic resonance (MR) scans of the phantom, image import, MR‐CT fusion, target contouring, treatment plan creation (seven isocenters, 405 beams/fraction, 27 Gy in 3 fractions), plan review/approval, secondary dose calculation, QA plan creation (coronal plane), QA delivery, and treatment plan delivery were performed. The QA plan was delivered using SRS MapCHECK (Sun Nuclear Corp., Melbourne, FL, USA) in SNC Patient software (version 8.6.0.76, Sun Nuclear Corp.) (Figure 19a) and 2D measured dose was compared with 2D calculated dose using gamma criteria of a dose difference of 2% and distance‐to‐agreement of 1 mm. The clinical treatment plan was delivered on the MAX HD phantom with an EBT3 film (coronal plane) in the film cube (Figure 19b). After 24 h, the film was scanned with 300 dpi on a scanner (EPSON 10000XL) and using ImageJ software, dose to the center of the target was read with dose calibration applied. The tolerances were set to ≥ 90% of a gamma passing rate with 2%/1 mm for a 2D plane and ± 5%^15^ for a point dose measurement.

## 3 RESULTS

Results for daily and monthly QA tests presented below were based on data over 4 months following commissioning of the system (at the time of writing this report).

### 3.1 Daily QA

#### 3.1.1 Warm‐up

*D1. Initialize*. Initialization was successfully completed.

*D2. Self‐check*. Self‐check passed.

*D3. MU delivery*. 6000 MU delivery was successfully completed.

#### 3.1.2 Dosimetry tests

*D4. Output constancy*. The baseline was set to 1.00 cGy/MU. The average daily output measured over 4 months was –0.80% ± 0.61% (range: –1.91%–0.69%) and was well within the tolerance of ± 3% of the set baseline.

*D5. Dose rate*. The average dose rate measured over 4 months for the nominal dose rate of 1500 MU/min was 1500.5 MU/min ± 40.6 MU/min (range: 1450.0–1562.0 MU/min) and was within the tolerance (1395.0–1575.0 MU/min).

#### 3.1.3 Mechanical tests

*D6. Collimator selection*. All eight collimators were tested to be functional over 4 months.

**FIGURE 20:**
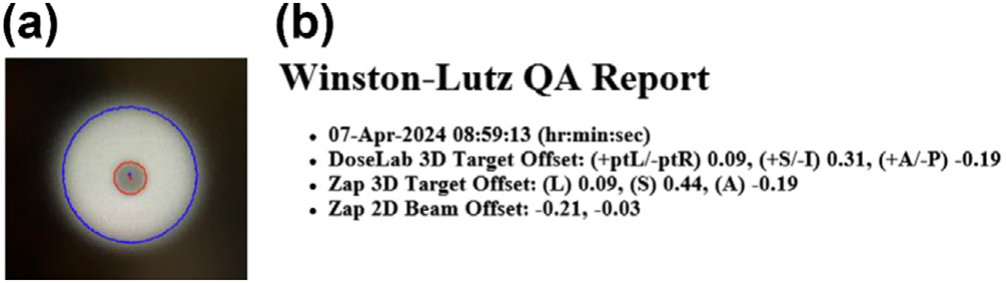
(a) MV image and (b) results for the steel ball test as analyzed on the UI. On the MV image, the red dot represents the mechanical isocenter (center of the steel ball) and the blue dot indicates the radiation center (center of the MV beam). MV, megavoltage, UI, user interface.

*D7. Steel ball test*. Figure 20 shows an example of the steel ball test result. Average 3D target offsets in left‐right, superior‐inferior, and anterior‐posterior directions over 4 months were 0.08 mm ± 0.06 mm (range: –0.05–0.19 mm), 0.53 mm ± 0.07 mm (range: 0.40–0.67 mm), and –0.11 mm ± 0.06 mm (range: –0.26–0.02 mm) in order. The offsets in all directions were within the tolerance (± 0.75 mm).

#### 3.1.4 Safety tests

*D8. Safety*. All safety features and interlock systems were functional over 4 months.

#### 3.1.5 Imaging tests

**FIGURE 21:**
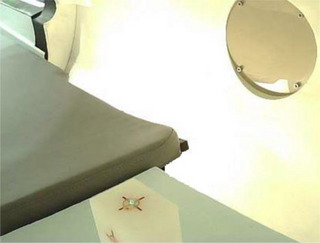
Coincidence of the laser (green) and the anterior marker for positioning/repositioning accuracy check using kV imaging. kV, kilovoltage.

*D9. Positioning/repositioning*. The laser was at the anterior and lateral markers (Figure 21) and the coincidence between the laser and the markers was within the tolerance (1 mm).

**FIGURE 22:**
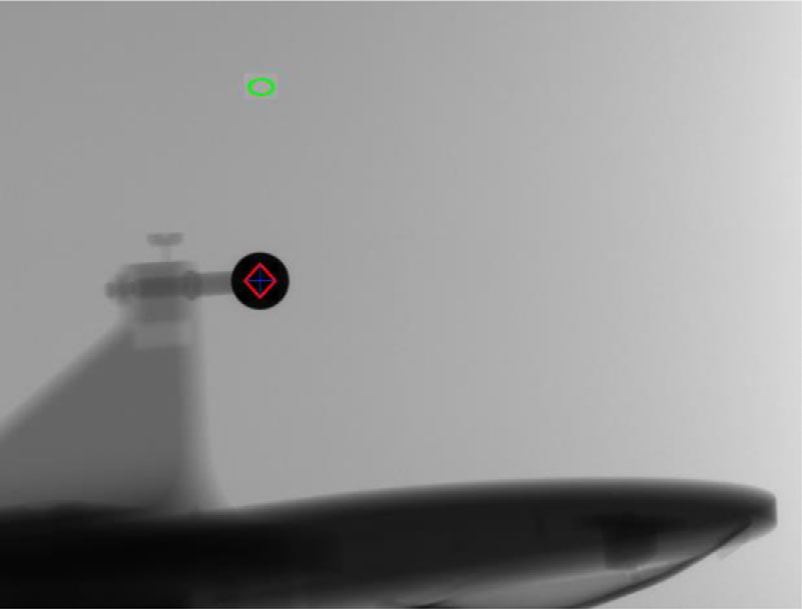
kV image of the steel ball for imaging and treatment coordinate coincidence check. kV, kilovoltage.

*D10. Imaging and treatment coordinate coincidence (single gantry position)*. For the North Pole position, the electronic crosshairs (imaging isocenter) were within ± 3.5 pixels from the center of the steel ball (treatment isocenter) on kV images (tolerance) for 4 months (Figure 22).

#### 3.1.6 Other test(s)

*D11. Auxiliary parameters*. Baseline values for water temperature, water flow, SF6 pressure, machine air pressure, and compressor air pressure were 38.0°C, 12.15 g/m, 29 psi, 100 psi, and 140 psi, respectively. Corresponding average measured values were 38.0°C ± 0.0°C, 11.80 g/m ± 0.09 g/m (range: 11.7–12.0 g/m), 29.46 psi ± 0.44 psi (range: 28.4–29.9 psi), 104.17 psi ± 3.51 psi (range: 100.0–110.0 psi) and 149.92 psi ± 8.88 psi (range: 133.0–160.0 psi). The measurements over 4 months were all within the tolerances (Table 1).

### 3.2 Monthly QA

#### 3.2.1 Dosimetry tests

*M1. Output constancy*. The baseline was set to 1.00 cGy/MU. The average monthly output measured over 4 months was 0.65% ± 0.78% (range: –0.24%–1.65%) and was within the tolerance of ± 2% from the baseline.

*M2. Beam energy constancy*. The baseline value was 0.3801. The average measured beam energy was –0.16% ± 0.34% with a range from –0.55% to 0.24% which was within the tolerance (± 1% from the baseline) for 4 months.

*M3. Backup monitor chamber constancy*. The average secondary MU monitored over 4 months was –0.85% ± 0.08% (range: –0.93%–(–)0.75%) for 500 MU and was within the tolerance (± 2% from the nominal MU).

**FIGURE 23:**
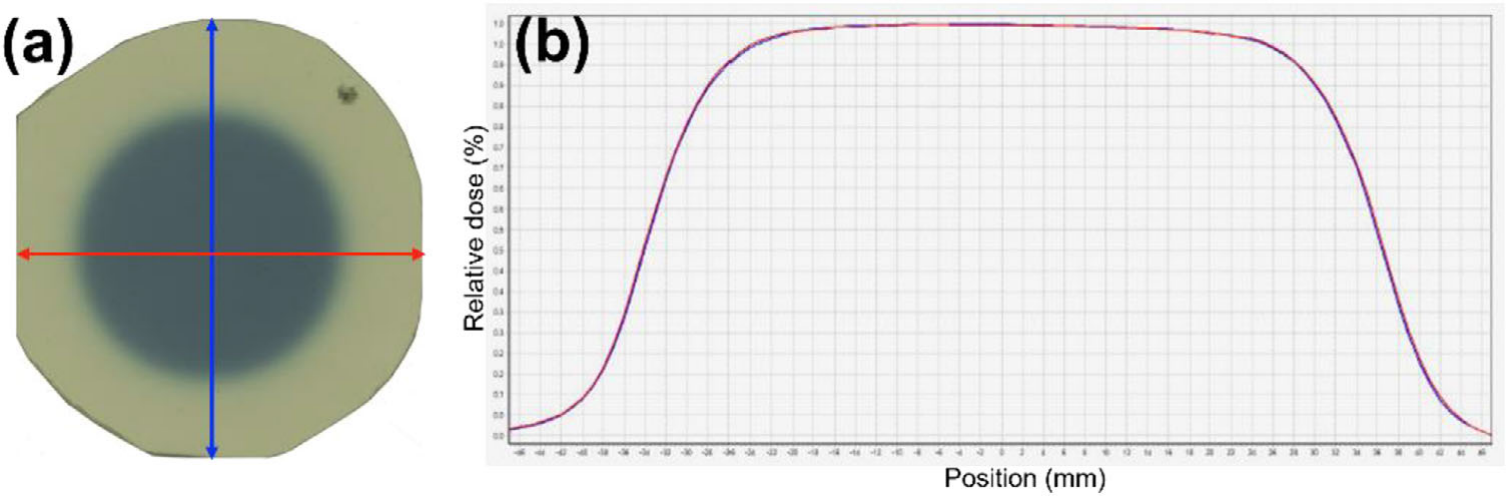
(a) Irradiated film and (b) profiles of wheel (red) and ortho (blue) planes for the 25 mm collimator.

*M4. Beam profile constancy*. Figure 23a,b show the irradiated film and profiles of wheel (red) and ortho (blue) planes for the 25 mm collimator, respectively. Baseline symmetries for wheel and ortho planes were 1.6% and 1.1%, respectively. Average symmetries for wheel and ortho planes over 4 months were 1.05% ± 0.66% (range: 0.3%–1.6%) and 1.18% ± 0.90% (range: 0.1%–2.3%), respectively and were within the tolerance (± 2% from the baseline).

#### 3.2.2 Mechanical tests

*M5. Laser alignment*. The center of the laser was aligned to the isocenter (deviation of 0 mm) for 4 months and the laser alignment was within the tolerance (1.5 mm).

*M6. Gantry angular accuracy*. For 4 months, average measured angles for Home, North Pole, Patient Right, South Pole, and Patient Left positions of the linac were 45.38° ± 0.15° (range: 45.2°–45.5°), 0.40° ± 0.34° (range: 0.2°–0.9°), 89.70° ± 0.18° (range: 89.5°–89.9°), 0.5° ± 0.22° (range: 0.2°–0.7°), and 89.65° ± 0.39° (range: 89.1°–90.0°), respectively. The measurements were within the tolerance (± 1°).

*M7. Treatment couch position indicators*. For 4 months, average measured table positions (50 mm from isocenter) in table left, right, out, in, down, and up directions were –49.75 mm ± 0.29 mm (range: –50.0–(–)49.5 mm), 50.00 mm ± 0.00 mm, –50.00 mm ± 0.00 mm, 50.00 mm ± 0.00 mm, –50.58 mm ± 0.43 mm (range: –51.0–(–)50.0 mm), and 50.38 mm ± 0.48 mm (range: 50.0–51.0 mm), respectively. The measurements were within the tolerance (± 1 mm).

**FIGURE 24:**
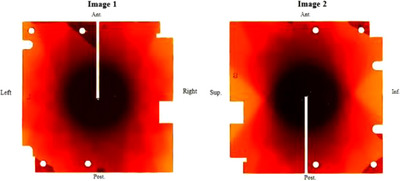
Irradiated orthogonal films evaluating mechanical accuracy in left‐right and anterior‐posterior directions, as well as in superior‐inferior and anterior‐posterior directions.

*M8. E2E testing*. Figure 24 shows the irradiated orthogonal films indicating differences between radiation center and mechanical isocenter in anterior‐posterior and left‐right directions as well as in anterior‐posterior and superior‐inferior directions. The average difference combined for three directions over 4 months was 0.60 mm ± 0.17 mm (range: 0.35–0.74 mm) and was within the tolerance (0.95 mm).

#### 3.2.3 Safety tests

*M9. Collimator collision test*. For 4 months, the collision indicator in the UI was functional and the table motion inactivation was functional as well.

#### 3.2.4 Imaging tests

*M10. Imaging and treatment coordinate coincidence (four selected gantry positions)*. For Home, Patient Right, South Pole, Patient Left positions, the electronic crosshairs (imaging isocenter) were within ± 3.5 pixels from the center of the steel ball (treatment isocenter) on kV images (tolerance) for 4 months.

*M11. Scaling*. The average diameter measured on kV images for five linac positions (Figure 22) was 19.0 mm ± 0.1 mm (range: 18.8–19.1 mm) and was within the tolerance (± 1 mm).

**FIGURE 25:**
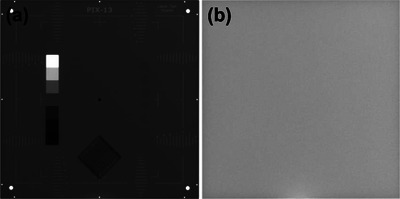
kV images of (a) PIX‐13 phantom and (b) uniform slab phantom for kV image quality check. kV, kilovoltage.

*M12*. kV *image quality*. kV images of the PXI‐13 phantom and the slab phantom are shown in Figure 25. Spatial resolution was 2.2 line pairs per mm (baseline). The numbers of visibly resolved circles and dynamic wedges (contrast) were 4 and 7 (baselines), respectively. Uniformity baseline was < ± 0.60% (center: 0.06%; 12 o'clock: –0.02%; 3 o'clock: –0.59%; 6 o'clock: 0.15%; 9 o'clock: 0.40%) and was within the tolerance (± 5% from the average value for 5 ROIs). Noise baseline was 0.00671. Over 4 months, spatial resolution and contrast remained the same as the baselines. For 4 months, average measured uniformity were 0.14% ± 0.06% (range: 0.06%–0.21%), –0.02% ± 0.04% (range: –0.07%–0.03%), –0.63% ± 0.05% (range: –0.68%–(–)0.59%), 0.12% ± 0.06% (range: 0.04%–0.18%), and 0.39% ± 0.02% (range: 0.35%–0.41%) for center, 12 o´clock, 3 o´clock, 6 o´clock', and 9 o´clock positions, respectively which were within the tolerance (± 5% from the average value). Average noise measured over 4 months was –0.33% ± 0.27% (range: –0.62%–(–)0.24%) and was also within the tolerance (± 5% from the baseline).

### 3.3 Annual QA

#### 3.3.1 Dosimetry tests

*A1. Beam profile constancy*. Symmetry and flatness for the wheel plane were 1.62% and 4.22% and corresponding values for the ortho plane were 0.81% and 3.89% (commissioning data). Measured field size for eight collimators is presented in Table 2 (commissioning data).

**TABLE 2:** Field size and output factors for eight collimators (annual QA).

	Field size (mm)		Output factor	
Collimator size (mm)	Wheel plane	Ortho plane	microDiamond	MicroSilicon
4	4.16	4.16	0.7059	0.7228
5	5.16	5.16	0.7830	0.7853
7.5	7.52	7.52	0.8836	0.8847
10	10.01	10.00	0.9274	0.9247
12.5	12.47	12.49	0.9537	0.9520
15	14.99	14.96	0.9675	0.9660
20	19.95	19.92	0.9839	0.9864
25	24.94	24.92	1.0000	1.0000

Abbreviation: QA, quality assurance.

*A2. Beam quality*. PDD at 10 cm for the 25 mm collimator was 40.54% (commissioning data).

*A3. Output calibration*. Machine output was calibrated to 1.00 cGy/MU (nominal output).

*A4. Output factors*. Measured output factors are presented in Table 2. Absolute differences between microDiamond and microSilicon detectors were < 0.03 and the largest difference was observed for the 4 mm collimator. The output factors measured using the microDiamond were entered in our Zap‐X TPS (commissioning data).

*A5. MU linearity*. MU linearity was 0.07%, 0.01%, –0.01%, 0.00%, and 0.00% for 10, 50, 100, 500, and 1000 MU, respectively, and was within the tolerance (± 2%).

*A6. Output constancy versus gantry angle*. Output variations were 0.00%, 1.00%, –4.28%, and 0.62% for North Pole, Patient Right, South Pole and Patient Left, respectively, and were within the tolerance (± 1% of the value at North Pole) except for the South Pole position. The largest variation (–4.28%) in South Pole was attributed to couch attenuation.

#### 3.3.2 Mechanical tests

**FIGURE 26:**
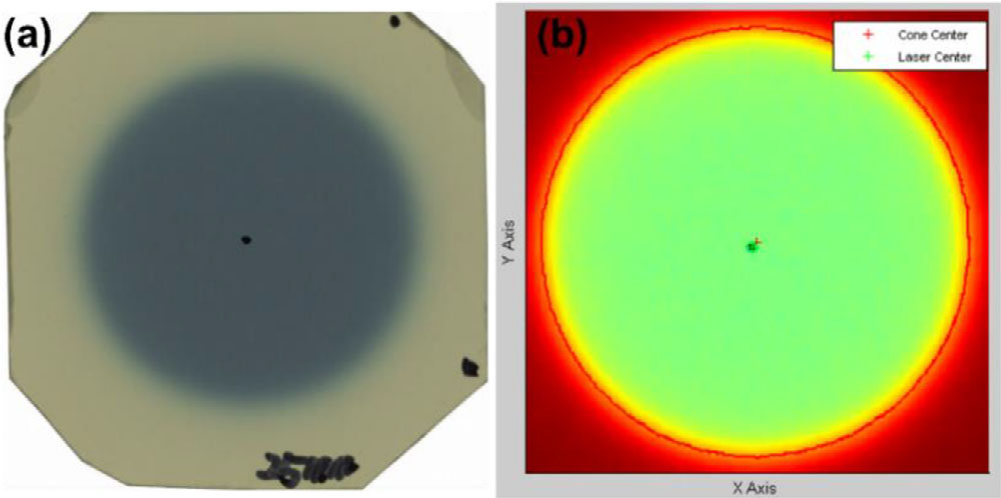
(a) Irradiated film and (b) film analysis for collimator wheel positioning accuracy check.

*A7. Positioning accuracy of the collimator wheel*. Figure 26a,b show the irradiated film with the laser center marked and film analysis displaying the laser center (isocenter, green) and the collimator center (red) for the 25 mm collimator, respectively. Distances from the laser center to the collimator center were 0.330, 0.231, 0.298, 0.314, 0.410, 0.407, 0.241, and 0.356 mm for 4, 5, 7.5, 10, 12.5, 15, 20, and 25 mm collimators, respectively. The measurements were within the tolerance (0.5 mm).

**FIGURE 27:**
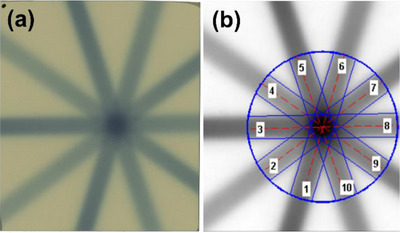
(a) Irradiated film and (b) film analysis for the star shot measurement.

*A8. Coincidence of radiation and mechanical isocenter*. The irradiated film and film analysis for the star shot measurement are shown in Figure 27. The measured minimum tangent circular radius was 0.18 mm (baseline).

*A9. Tabletop sag*. The tabletop sag was 2 mm (baseline) with 202.5‐lb phantoms.

#### 3.3.3 Safety tests

*A10. Radiation survey for leakage*. Results for the radiation survey are presented in Table 3. Leakage radiation measured in 14 locations for the five linac positions was all within the tolerance (2 mR/hr).

**TABLE 3:** Leakage radiation measurement results at various positions around the Zap‐X system for five selected linac positions (annual QA).

	Instantaneous exposure (mR/hr)				
Detector location	Home	North pole	Patient right	South pole	Patient left
1	1.49	0.82	1.68	0.07	1.58
2	0.10	0.62	0.22	0.42	0.14
3	0.12	0.49	0.19	0.62	0.11
4	0.08	0.18	0.12	0.25	0.06
5	0.07	0.11	0.10	0.11	0.04
6	0.52	0.11	0.08	0.17	0.66
7	0.53	0.43	0.05	0.15	0.88
8	0.11	0.15	0.25	0.22	0.46
9	0.75	0.21	0.95	0.48	0.10
10	0.44	0.20	0.40	0.15	0.11
11	0.03	0.10	0.04	0.11	0.07
12	0.07	0.22	0.07	0.14	0.15
13	0.11	0.19	0.11	0.38	0.37
14	0.11	0.13	0.12	0.32	0.36

*Note*: Measurements were made in a full scatter condition (head and body phantoms on the table with maximum collimator size of 25 mm).

Abbreviations: linac, linear accelerator; QA, quality assurance.

*A11. Emergency procedures/features*. Reviewing and performing emergency procedures were completed, and all emergency features were functional.

#### 3.3.4 Imaging tests

*A12. Beam quality/energy*. Measured energy was 88.53 kV ± 1.35 kV (–1.64%) for 90 kVp and was within the tolerance (± 5% from the nominal kV_p_).

*A13. Imaging dose*. Measured imaging dose was 6.64 µGy ± 0.04 µGy (baseline).

#### 3.3.5 Other test(s)

**FIGURE 28:**
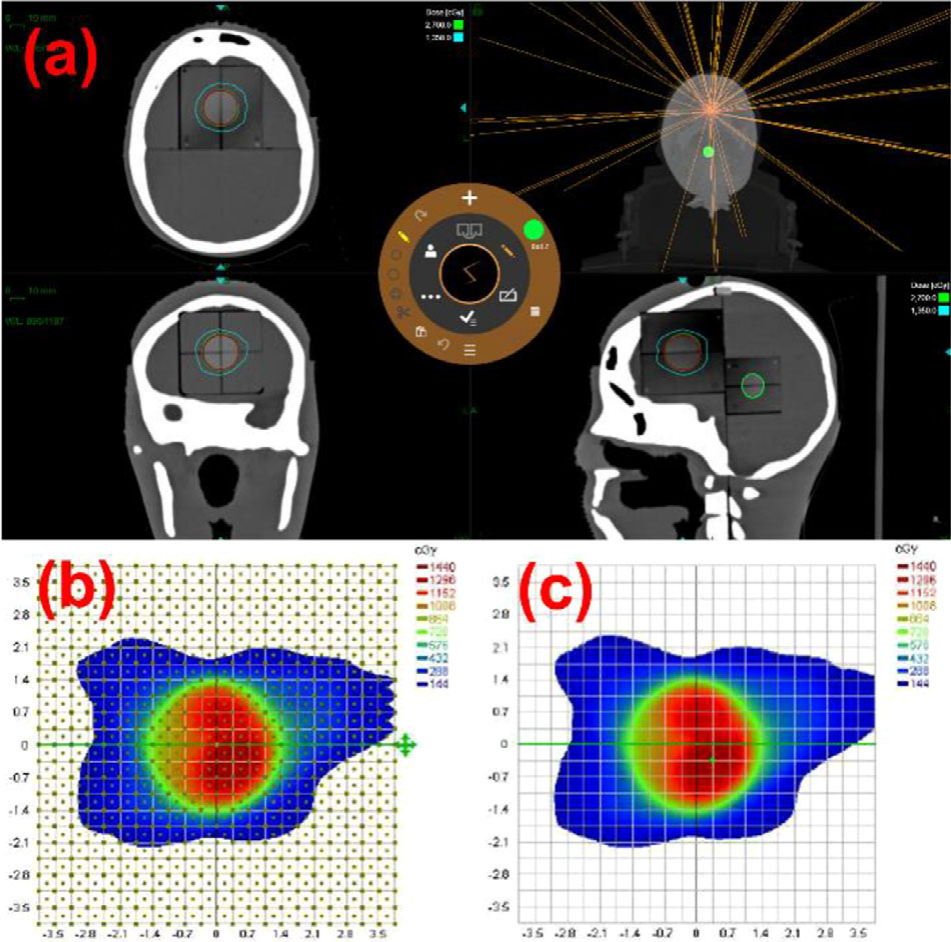
(a) Treatment plan created on the MAX HD SRS phantom, (b) measured 2D dose (coronal plane) and (c) calculated 2D dose (coronal plane) for a QA plan (complete end‐to‐end testing). QA, quality assurance.

*A14. Complete E2E testing*. Figure 28a shows the treatment plan created on the MAX HD SRS phantom in Zap‐X TPS. Figure 28b,c display measured 2D dose and calculated 2D dose for the QA plan, respectively. The gamma passing rate (%) was 100% with gamma criteria of 2%/1 mm. Measured point dose at the center of the target on the irradiated film was 0.99% of the dose calculated in Zap‐X TPS. The measurements were within the tolerances (≥ 90% with 2%/1 mm for a 2D plane and ≤ ± 5% for point dose).

## 4 DISCUSSION

Excluding a few tests, the principles of most daily QA tests and their tolerances recommended in the TG‐142 report were adopted for the Zap‐X system (Table 4). Among the daily mechanical tests as outlined in the TG‐142 recommendations, ODI and collimator size indicator were not implemented. ODI or a collimator size indicator is not verified because there is an absence of ODI and jaws in the Zap‐X system. Since the laser is not used for patient setup, a laser alignment check is performed monthly in our clinic. Collision interlocks of an imaging system in TG 142 were also not implemented because the kV imaging system inside the Zap‐X unit has no collision interlocks.

**TABLE 4:** A summary of QA tests not adopted from AAPM TG 142 or MPPG 8.b and additional tests added from TG 135 or the vendor's recommendations.

	QA tests not adopted from TG 142^13^ or MPPG 8.b^15^	QA tests added from TG 135^16^ or the vendor^1^
**Daily**	**Mechanical tests**	**Warm‐up**
	Distance indicator (ODI)	D1. Initialize
	Collimator size indicator	D2. Self‐check
	**Imaging tests**	D3. MU delivery: 6000 MU
	Collision interlocks	**Mechanical tests**
		D6. Collimator selection: all 8 collimators
		**Safety tests**
		D8. Interlocks: proximity sensors, HV off button
		**Other test(s)**
		D11. Auxiliary parameters: compressor air pressure
**Monthly**	**Mechanical tests**	**Mechanical tests**
	Light/radiation field coincidence	M8. E2E testing
	Distance check device for lasers compared with front pointer	
	Collimator angle indicators	
	Jaw position indicators	
	Crosshair centering (walkout)	
**Annual**	**Dosimetry tests**	**Mechanical tests**
	Output constancy versus dose rate	A7. Positioning accuracy of the collimator wheel: all eight collimators
	**Mechanical tests**	**Safety tests**
	Collimator rotation isocenter	A10. Radiation survey for leakage
	Gantry rotation isocenter	
	Couch rotation isocenter	
	Table angle	
	Table travel maximum range movement in all directions	

Abbreviations: AAPM, American association of physicists in medicine; E2E, end to end; HV, high voltage; MPPG, medical physics practice guideline; MU, monitor unit; ODI, optical distance indicator; QA, quality assurance; TG, task group.

Several tests specific to Zap‐X were incorporated into the daily QA. These include initialization, self‐check, 6000 MU delivery (warm‐up), collimator selection (mechanical test), interlock systems (proximity sensors and HV off button) (safety tests), and compressor air pressure monitoring (other test) (Table 4). Initialization is mandatory as the system does not permit beam‐on without it. Unlike other linacs, ZAP‐X utilizes a rotating wheel that houses all eight collimators requiring daily verification of collimator selection. The proximity sensors and HV off button are specific to the Zap‐X and must be tested daily for safety. The treatment door operates via air pressure from the compressor and hence, the pressure level must be checked daily.

Some monthly QA tests from TG 142 were not implemented due to the features of the Zap‐X. The tests encompass light/radiation field coincidence, distance check device for lasers compared with front pointer, collimator angle indicators, jaw position indicators, and crosshair centering (mechanical tests) (Table 4). The Zap‐X does not have a light field, ODI, collimator angle, nor jaws. Therefore, the tests mentioned above were not necessary for Zap‐X. Light and radiation field coincidence cannot be checked but field (collimator) size should be checked at least annually.

One test added in our monthly QA was taken from the TG‐135 recommendations (QA tests for CK). Because of similarities between Zap‐X and CK systems (e.g., kV imaging‐based pre‐treatment verification and intrafraction monitoring), E2E testing (mechanical test) from TG 135 was adopted (Table 4). Since daily self‐check includes testing the collimator collision sensor, the collimator collision test was added in monthly QA.

For similar reasons, some annual QA tests from TG 142 were not adopted (Table 4). The Zap‐X has only one nominal dose rate (1500 MU/min) and hence, output constancy versus dose rate (dosimetry test) was excluded from annual QA. Collimator, gantry, and couch rotations as well as table angle (mechanical tests) are not part of the features of the Zap‐X and were also excluded. Additionally, table travel maximum range movement in all directions (mechanical test) was not included in annual QA. The table can move > 10 cm in each direction from the isocenter (except the anterior direction which is limited to approximately 9 cm). For typical treatment to a brain lesion, table movement of 18 cm (= 9 cm × 2) from one isocenter to another in each direction is not required. Therefore, the maximum travel range of table was not incorporated into the annual checks but should be noted during commissioning efforts.

Two additional annual QA tests were implemented (Table 4). The collimator wheel, specific to Zap‐X system, requires an annual check for positioning accuracy (mechanical test). The unit is self‐shielded, but the proximity of operator console necessitates an annual measurement of leakage radiation levels (safety test).

Most QA tests implemented for the Zap‐X require the vendor provided QA devices (e.g., F‐bracket, front pointer, star shot fixture, etc.) primarily due to limited physical access to the isocenter, no ODI/light field/external laser, and limitation of table motion control. Nonetheless, there might be better and/or more efficient ways of performing some of the QA tests presented here by using different QA tool(s) (e.g., 3D‐printed mount) and/or by automated analysis (i.e., imaging QA). Continuous QA process improvement will be warranted in the future. With software and hardware upgrades from the vendor, it is expected that QA tests will be done more efficiently. For example, in the version of DP‐1010, the mechanical test for couch (M7 test) and kV imaging tests (D9‐D10, M10‐M12, and A12‐A13 tests) can be performed in Daily QA mode, rather than in service mode.

## 5 CONCLUSION

This report has comprehensively detailed the daily, monthly, and annual QA methods for the Zap‐X system implemented in our clinic following the recommendations set forth in TG 142. The QA program developed and discussed here should serve to provide guidance to Zap‐X teams in other Neurosurgery centers or Radiation Oncology clinics in establishing and maintaining their QA programs, especially in the absence of AAPM endorsed guidelines.

## AUTHOR CONTRIBUTIONS

Yongsook C. Lee designed the concept, performed all measurements, and wrote the initial version of the manuscript. Ranjini Tolakanahalli, D Jay Wieczorek, and Alonso N. Gutierrez provided scientific reviews and contributed to the final manuscript. Minesh P. Mehta, Michael W. McDermott and Rupesh Kotecha contributed to the final manuscript.

## CONFLICT OF INTEREST STATEMENT

Yongsook C. Lee: Honorarium from Zap Surgical Systems, Inc.; Ranjini Tolaknahalli: Honoraria from MIM Software Inc. and GE Healthcare; Minesh P. Mehta: Consulting fees from AIQ, Telix, Novacure, Zap Surgical Systems, Inc., Mevion, Xoft, and Kazia Therapeutics and Stock in Chimerix; Michael W. McDermott: Consultant for Stryker Co. and DiendeMedical; Rupesh Kotecha: Personal fees from Accuray Inc., Elekta AB, ViewRay Inc., Novocure Inc., Elsevier Inc., Brainlab, Kazia Therapeutics and Castle Biosciences, and institutional research funding from MedtronicInc., Blue Earth Diagnostics Ltd., Novocure Inc., GTMedical Technologies, AstraZeneca, Exelixis, ViewRayInc., Brainlab, Cantex Pharmaceuticals, and KaziaTherapeutics; Alonso N. Gutierrez: Honoraria from ViewRay, Inc., Elekta AB, CQ Medical, Inc, and IBA AB; Advisory Board for IBA AB.

## Data Availability

Data are not shared.
